# EPA’s DSSTox database: History of development of a curated chemistry resource supporting computational toxicology research

**DOI:** 10.1016/j.comtox.2019.100096

**Published:** 2019-11-01

**Authors:** Christopher M. Grulke, Antony J. Williams, Inthirany Thillanadarajah, Ann M. Richard

**Affiliations:** a National Center for Computational Toxicology, Office of Research & Development, US Environmental Protection Agency, Mail Drop D143-02, Research Triangle Park, NC 27711, USA; b Senior Environmental Employment Program, US Environmental Protection Agency, Research Triangle Park, NC 27711, USA

**Keywords:** DSSTox, QSAR, Computational toxicology, Environmental science, Chemistry database, Data quality, Structure curation

## Abstract

The US Environmental Protection Agency’s (EPA) Distributed Structure-Searchable Toxicity (DSSTox) database, launched publicly in 2004, currently exceeds 875 K substances spanning hundreds of lists of interest to EPA and environmental researchers. From its inception, DSSTox has focused curation efforts on resolving chemical identifier errors and conflicts in the public domain towards the goal of assigning accurate chemical structures to data and lists of importance to the environmental research and regulatory community. Accurate structure-data associations, in turn, are necessary inputs to structure-based predictive models supporting hazard and risk assessments. In 2014, the legacy, manually curated DSSTox_V1 content was migrated to a MySQL data model, with modern cheminformatics tools supporting both manual and automated curation processes to increase efficiencies. This was followed by sequential auto-loads of filtered portions of three public datasets: EPA’s Substance Registry Services (SRS), the National Library of Medicine’s ChemID, and PubChem. This process was constrained by a key requirement of uniquely mapped identifiers (i.e., CAS RN, name and structure) for each substance, rejecting content where any two identifiers were conflicted either within or across datasets. This rejected content highlighted the degree of conflicting, inaccurate substance-structure ID mappings in the public domain, ranging from 12% (within EPA SRS) to 49% (across ChemID and PubChem). Substances successfully added to DSSTox from each auto-load were assigned to one of five *qc_levels*, conveying curator confidence in each dataset. This process enabled a significant expansion of DSSTox content to provide better coverage of the chemical landscape of interest to environmental scientists, while retaining focus on the accuracy of substance-structure-data associations. Currently, DSSTox serves as the core foundation of EPA’s CompTox Chemicals Dashboard [https://comptox.epa.gov/dashboard], which provides public access to DSSTox content in support of a broad range of modeling and research activities within EPA and, increasingly, across the field of computational toxicology.

## 1. Background

The US Environmental Protection Agency’s (EPA) Distributed Structure-Searchable Toxicity (DSSTox) database was publicly launched in early 2004 as a manually curated aggregation of more than 7000 chemical substances spanning half a dozen chemical inventories of interest to EPA and environmental toxicology researchers [1].^1^ These inventories included chemicals tested for rodent carcinogenicity and mutagenicity, fathead minnow aquatic toxicity, and estrogen receptor binding, later expanding to include lists of high-production volume (HPV) chemicals, disinfection by-product chemicals, chemically-indexed microarray experiments, and chemicals for which EPA had conducted risk assessments (for more detail on early published lists, see, e.g., [2]). A major goal, from the start, was to establish accurate linkages of chemical structures to source substance identifiers (typically Chemical Abstract Service Registry Numbers - CAS RNs, and chemical names), thereby providing high quality associations of chemical structures to toxicity and bioactivity data, as well as to chemical lists of regulatory importance. A secondary goal was to use chemical structures to enable chemistry-based cross-referencing of “siloed” EPA chemical lists. Most lists with associated bioactivity and property data of interest to environmental scientists that were available at that time, including regulatory lists, Internet resources and the scientific literature, were indexed either by highly variable and error-prone chemical names only, or by names and CAS RNs only. Chemical structures, in turn, are universally recognized as the *lingua franca* of chemistry, can be uniquely rendered with publicly available formats, and are required inputs for any type of structure-based predictive modeling, which was playing an increasing role in filling data gaps in environmental hazard and risk assessments. Hence, many independent groups repeatedly faced the challenge of assigning chemical structures directly to data and lists using only the source-provided name and/or CAS RN identifiers. DSSTox curators recognized from the earliest days, as have many others before and since, that frequently encountered errors in associations of chemical names and CAS RN to data introduces errors and uncertainty into the assignment of chemical structures to data, which directly undermines structure-based prediction models. Thus, the DSSTox project aims were two-fold: 1) to provide and promote the creation of high quality, standardized chemical structure-data files in support of quantitative structure-activity relationship (QSAR) modeling of toxicity; and 2) to enable structure-based cross-referencing and searching of previously siloed chemical lists across the environmental chemical research and regulatory landscape [3,4].

Subsequent to the initial launch of DSSTox, two much larger chemical l databases entered the public realm with a structure-centered focus: PubChem [https://pubchem.ncbi.nlm.nih.gov/], a user-depositor model hosting source-defined substance-bioassay data and fully downloadable, launched in September 2004 [5,6] and ChemSpider [http://www.chemspider.com/], a chemical structure aggregator database, that is searchable but not fully downloadable, launched in March 2007 [7]. These databases quickly expanded to provide searchability across a few million chemicals and today each exceeds tens-of-millions of compounds (Jan 29, 2019: PubChem 97 million; ChemSpider 71 million).^2^ A distinguishing feature of both PubChem and ChemSpider is a primarily structure-centric data model with a corresponding focus on chemical structure normalization and standardization [8,9]; “PubChem,”). Hence, chemical structure serves as a key index, whereas fewer constraints are placed on other generic chemical identifiers, such as CAS RN and names, in relation to structure. This means that whereas structures are unique in the database, there can be multiple incidences of a name or CAS RN linked to different structures (i.e., names and CAS RN are not uniquely mapped to structure), and multiple hits for common chemicals can result (see Suppl. Material, Example 1).

The features that initially distinguished DSSTox from these larger database projects -a focus on establishing quality structure-data-list associations for environmental chemicals using manual curation to resolve conflicts identifiers - also limited its growth. EPA’s National Center for Computational Toxicology (NCCT) was formed in 2005 with a mandate to implement advances in screening technologies and computational approaches to transform toxicology into a more cost-effective, high-throughput enterprise later articulated by the National Research Council [10]. Although the DSSTox project was incorporated into NCCT at that time, the need for a much larger chemical database spanning the global environmental chemistry landscape became increasingly apparent. EPA’s ACToR database was released in 2009 with the express goal of aggregating a larger universe of environmental chemicals associated with toxicology and regulatory activities [11]. Since most such lists did not contain chemical structures, and given the widespread use of CAS RNs across the published scientific literature, Internet resources, and the chemical regulatory domain, ACToR adopted CAS RN as its primary aggregator and unique database index. Chemical names and synonyms were collected as secondary identifiers with no enforcement of uniqueness. The ACToR project, by means of auto-loading of CAS RN-indexed lists, successfully processed and cross-referenced hundreds of lists and hundreds of thousands of CAS RN-Name-substances [12]. ACToR incorporated the full DSSTox CAS RN-Name-structure content available at the time but lacked chemical structures and DSSTox manual curation review for the major portion of its content.

In 2007, shortly after its formation, NCCT launched its signature research program - the ToxCast high-throughput screening (HTS) project [13]. At around the same time, EPA joined as a major partner in the Tox21 HTS cross-federal Agency project, along with the National Institutes of Health’s (NIH) National Toxicology Program (NTP) and National Center for Advancing Translational Sciences (NCATS) [14]. In the early phases of ToxCast, DSSTox and ACToR each played a major role in nominating thousands of CAS RN-indexed chemicals for acquisition and testing based on various relevancy factors pertaining to data availability, environmental occurrence, and regulatory and research interest. From the start, however, the DSSTox project was tasked with curating and registering all physical samples entering the ToxCast and Tox21 testing library to ensure high-quality structure-data annotations. Hence, the DSSTox database grew to encompass the entirety of EPA’s ToxCast library and the larger multi-Agency Tox21 library, the latter exceeding 8500 unique test substances by 2011 [15]. At the same time, EPA’s computational toxicology research programs were broadening in scope across diverse data landscapes and spanning ever larger domains of uncurated chemistry pertaining to animal toxicity, target-based activity, endocrine disruption, consumer product usage, and environmental exposure (see, e.g., Cohen Hubal et al. [16] and Egeghy et al. [17]). The central role of chemistry in providing structure-data linkages and supporting structure-based modeling reinforced the need to deliver a single, high quality chemical database to service EPA’s computational toxicology research programs as well as EPA’s broader research and regulatory needs moving forward.

By 2013, DSSTox had reached a point where manual curation and maintenance of multiple tables of text-based information for upwards of 24,000 substances, the majority linked to structure-data (SD) format files, constituted an unsustainable model for future expansion. However, more than a decade’s worth of experience manually curating public chemical lists and sets of commercially procured chemicals by a small team of EPA researchers and support staff had provided a wealth of insight into the nature and extent of errors encountered in the curation of chemistry in the public domain, underscoring the critical need for such curation. This experience, coupled with insights pertaining to the nature of CAS RN-substance-structure linkages, substantially informed the design and implementation of the second generation of DSSTox (DSSTox_V2). The first step was to convert the original DSSTox tabular and structure content (DSSTox_V1) to a MySQL database supported by modern cheminformatics structure processing tools, both commercially and publicly available. DSSTox_V2 subsequently underwent a series of quality-filtered auto-load list expansions, adding content from several public chemical databases to the original DSSTox_V1 content. This expansion was both enabled and constrained by the enforcement of a rule requiring a unique mapping of CAS RN-substance to structure, i.e. requiring that a single CAS RN (or a DSSTox CAS RN-like identifier when a CAS RN is unavailable) be uniquely assigned to each distinct substance and, wherever possible, to a unique structure. This initial build succeeded in expanding DSSTox_V2 to more than 740,000 chemicals assigned to five quality curation levels. Tens of thousands of new chemicals have since been added through a strategic combination of auto-loads, defined processes, and manual curation.

EPA’s CompTox Chemicals Dashboard (i.e., Dashboard), launched publicly in 2016, was built on the foundation of DSSTox_V2 and is the primary vehicle for providing public access to DSSTox’s chemical substance-structure database and indexed lists [https://comptox.epa.gov/dashboard] [18]. The Dashboard and associated outreach efforts have greatly expanded the reach of the DSSTox project to support a broad and growing range of data-gathering, modeling, and research activities within and outside of EPA. Within EPA, these include non-targeted analysis (NTA) mass-spectroscopy (MS) research, QSAR prediction of physicochemical property and toxicity endpoints, structure-based read-across, text-mining, access to ToxCast and Tox21 HTS data, support for external links, and various means for batch searching and exporting chemical lists, along with list overlaps, properties and annotations (see, e.g., Sobus et al. [19], McEachran et al. [20], Kamel Mansouri et al. [21], Helman et al. [22], Baker et al. [23]). Outside of EPA, examples of outreach and coordination include with PubChem, UNICHEM [https://www.ebi.ac.uk/unichem/], and support for NTA within the NORMAN Network [https://www.norman-network.net/].

The DSSTox_V2 data model currently constrains and governs all aspects of chemical curation, list registration, and registration of new substances, and is fully integrated with EPA’s ToxCast and Tox21 chemical management system. Most recently, it has been expanded to handle less well-defined chemical substances and emerging areas of chemical concern within EPA and the environmental toxicology community. The DSSTox project, from its inception to the present, has focused on the challenge of providing the most accurate chemical identifier associations possible for data and lists of importance to the environmental research and regulatory community. Consistent with that focus, DSSTox is the only publicly available chemical database that 1) is uniquely keyed to both CAS RN and structure, and 2) is supported by automated and expert manual list and substance curation. In the remainder of this article, we will describe the process by which DSSTox_V1 was updated to DSSTox_V2, along with details of the associated DSSTox_V2 data model that have enabled a significant expansion of DSSTox content, while retaining a strong focus on data quality. First, however, it is necessary to relate key insights underpinning the DSSTox data model that have proved essential to detecting and quantifying, for the first time, the large numbers of errors in public records, as well as to evaluating and ensuring the quality of identifier and data associations in registered DSSTox content.

## 2. A “CASe” of mistaken identity

The frequent incidence of conflicts in the association of chemical structures and identifiers in the environmental toxicology literature and public resources, and the ease and speed with which these errors propagate across the Internet, was recognized from the earliest days of the DSSTox project. These errors undermine the very foundation of efforts to accurately index chemical data and develop QSAR models: they degrade clarity of bioassay and test results, reduce confidence in prediction models, and affect data quality and integrity at every level of use. Much attention has been paid within the QSAR and cheminformatics community to the problems of detecting and correcting errors in the rendering and representation of chemical structures [24,25]. Without standardized, exchangeable representations for chemical structures, different representations of the same structure are treated as distinct and experimental data are incorrectly aggregated. A variety of public and commercial cheminformatics toolkits have been successfully applied to addressing this problem of structure normalization for both database storage [8,9,26] and modeling dataset preparation [24]. Far less attention has been paid to whether the structure and the associated chemical identifiers (CAS RN and name) are, on the one hand, internally consistent and, on the other hand, correctly mapped to the original source data or list. Although recent publications have reported attempts to address these issues using a consensus of public sources (see, e.g., Gadaleta, Lombardo, Toma, and Benfenati [27]), the identification and correction of mapping errors cannot be solved with automated methods relying on public resources alone (see, e.g., the WikiProject on CAS Validation [https://en.wikipedia.org/wiki/Wikipedia_talk:WikiProject_Chemistry/CAS_validation], a CAS-collaboration whose outcome was the Common Chemistry website [http://www.commonchemistry.org/]).

Hence, the DSSTox project faced the challenge of establishing standards of accuracy, or “truth” that could be used both to detect errors (i.e., deviations from truth) and offer a path to their resolution. CAS RNs came to play a pivotal role for many of the same reasons they served as a primary index in ACToR: CAS RNs permeate the public chemical-bioactivity and environmental literature, are often required to index chemicals in regulatory organizations in the US and worldwide, and are widely propagated across the Internet in public databases, chemical listings, and chemical supplier websites. In addition to their large public presence and usage, and more importantly for present purposes, CAS RNs are associated with definitive substance-structure records that exist within the commercially accessible Chemical Abstract Service (CAS) database [https://www.cas.org/products/scifinder; https://www.cas.org/products/stn].

A key insight gleaned in the process of manually curating over 20,000 chemicals for DSSTox_V1 was that it was indeed possible to establish and enforce a strict 1:1:1 mapping of CAS RN to a unique name and structure, primarily relying on available public resources but requiring limited use of the CAS database. The central problem is that the public body of CAS RN associations, represented and redistributed freely across the scientific literature and Internet, exist independently and without sanction or review from the owners of the proprietary CAS databases (the American Chemical Society). Hence, public CAS RN associations propagated across the Internet are replete with errors such that the uniqueness of CAS RN assignments can no longer be recognized or deconvoluted (see Suppl. Material, Examples 1–3). This gives rise to errors in which CAS RN, names and structure are incorrectly associated and the 1:1:1 CAS RN-Name-structure mapping rule within the public source list is often violated. Such conflicting information is common in lists or datasets loaded into DSSTox, and can be identified either within a set of source substance identifiers, confounding the source-substance identity, or can become apparent when the source-list identifiers are mapped to existing DSSTox content [28]. CAS RN mapping errors are most frequently encountered in public resources when chemicals with different parent/salt and stereo properties are conflated. Another common mapping error is caused by the public presence of “Deleted CAS RNs”; these are a type of CAS RN (sometimes referred to as “Other CAS RN”), numbering in the tens of thousands in the current DSSTox content, that have been listed (and publicly distributed) and later redacted in the CAS database and replaced by the currently listed “Active CAS RN” record for the substance. Hence, Deleted CAS RNs in the public domain create replicate mappings until they are identified and rerouted to the corresponding Active CAS RN.^3^

Given its public mission, DSSTox curation relies primarily on expert review and consensus of public sources of information to confirm identifiers for the vast majority of its registered substance records. However, in the minority of cases where public records are conflicting or non-existent and expert curators are unable to identify the valid mapping based on public sources, it is necessary to consult the proprietary CAS database to verify identities and resolve conflicts, particularly when registering high priority chemicals of public health concern and regulatory importance. Hence, DSSTox curation is guided by several rules: 1) a single Active CAS RN and unique name is assigned to each unique substance and, if no CAS RN record can be found, a unique DSSTox “NOCAS_#####” identifier is assigned to the substance^4^; 2) each DSSTox substance (name and CAS RN) is uniquely mapped to a structure, and when a unique structure cannot be assigned (e.g., non-stoichiometric mixtures, polymers, constitutional isomers), the substance name and CAS RN are uniquely mapped to a record without an associated chemical structure and a linkage to a closely related substance with a structure may be created; 3) a potential mapping error is detected whenever the 1:1:1 CAS RN-name-structure rule is violated; and 4) the only definitive source for determining the true association of a CAS RN with a substance and its associated structure is the aforementioned, commercially licensed CAS database accessed through CAS information tools such as SciFinder [https://www.cas.org/products/scifinder] or STN [https://www.cas.org/products/stn]. When applied to newly imported lists, the automated application of the first three rules allows for identification of many different categories of mapping errors, each of which gives rise to a defined manual curation process for resolution.

## 3. DSSTox_V2 data model

The DSSTox_V2 data model primarily centers on three types of chemical identifiers: CAS RNs, names (systematic, trivial, and source-provided), and chemical structures. Chemical names and CAS RN are designated as “generic substance” identifiers and are most closely associated with a test sample or a source-listed chemical and its associated data, whereas a unique structure is assigned to a defined substance whenever possible. These identifiers, as implemented in the DSSTox data model, are further described below.

### CAS RN

CAS RN found in the public domain are considered potentially valid if: 1) they have a defined 3-part numeric format (##…- ## - #); and 2) the last digit satisfies a checksum using a formula comprised of the previous digits [https://www.cas.org/support/documentation/chemical-substances/checkdig]. However, a CAS RN is deemed definitively valid ***only*** if it is confirmed to have a corresponding record within the commercial CAS database.^5^

### Chemical names

Chemical names fall into several categories that serve different functions in the data model: 1) a single “*preferred name*” is assigned to each unique DSSTox substance record and is generally chosen on the basis of sufficient chemical specificity, common usage, and concise length; 2) “*systematic names*” are based on formal chemical nomenclature rules (such as IUPAC [https://iupac.org/what-we-do/nomenclature/]), can usually be converted to the associated chemical structure using public and commercial name-to-structure tools, and may serve as a DSSTox preferred name or, otherwise, as a synonym for a substance; 3) “*synonyms*” are all other name-type identifiers loaded into the database and include “valid synonyms” (expert validated by DSSTox curators), “unique synonyms” (unique to a single substance record, i.e., no conflicts), and “ambiguous synonyms” (names commonly applied to multiple substances in public records) (see Suppl. Material, Example 4); and 4) “*source names*“ are imported with an original source list, without edit or correction, are internally stored as synonyms, and are used only for internal search-redirects to the preferred name.

### Structure

Structure in DSSTox is a representation of the substance in v3000 mol format [http://help.accelrysonline.com/ulm/onelab/1.0/content/ulm_pdfs/direct/reference/ctfileformats2016.pdf] that yields a unique InChIKey [https://inchi.info/inchikey_overview_en.html] (note, this is the default JChem InChIKey, not a Standard InChIKey). Versions of all cheminformatics softwares are updated frequently, so current versions in use at the time of publication are noted below. All structures in the database are managed primarily using cheminformatics functions from ChemAxon’s [https://www.chemaxon.com/] JChem Java API v18.5.0 [https://apidocs.chemaxon.com/jchem/doc/dev/java/api/] for structural conversion, image generation, and mass and formula calculations. The Indigo Toolkit v1.2.3 [http://lifescience.opensource.epam.com/indigo/] is employed to generate standard InChIs and InChIKeys. ACD/Labs Name Batch v2017.2 [http://www.acdlabs.com/products/draw_nom/nom/name/] is currently used to generate IUPAC and Index Names (based on IUPAC and CAS nomenclature rules) for all chemical structures. DSSTox structures are assigned to a substance record at the substance-specified level of stereochemistry (relative and absolute chiral stereo, E, Z, and mixture of E, Z double bond stereo can each be uniquely rendered in v3000 mol format^6^) for organics, salts, stoichiometric complexes, inorganics and organometallics. Previously, a structure was not assigned to a DSSTox substance in the case of most mixtures or chemicals of Unknown, Variable Composition, or of Biological Origin (UVCBs). More recently, however, Markush structure [https://en.wikipedia.org/wiki/Markush_structure] rendering of several mixtures and UVCBs has been enabled by integrating ChemAxon Markush Tools [https://chemaxon.com/products/markush-tools]. A Markush structure is, in essence, a structure-based query for representing a family of chemicals (*vide infra*), but given that they can be uniquely rendered, constitute a new type of DSSTox structure.

**Fig. 1.**
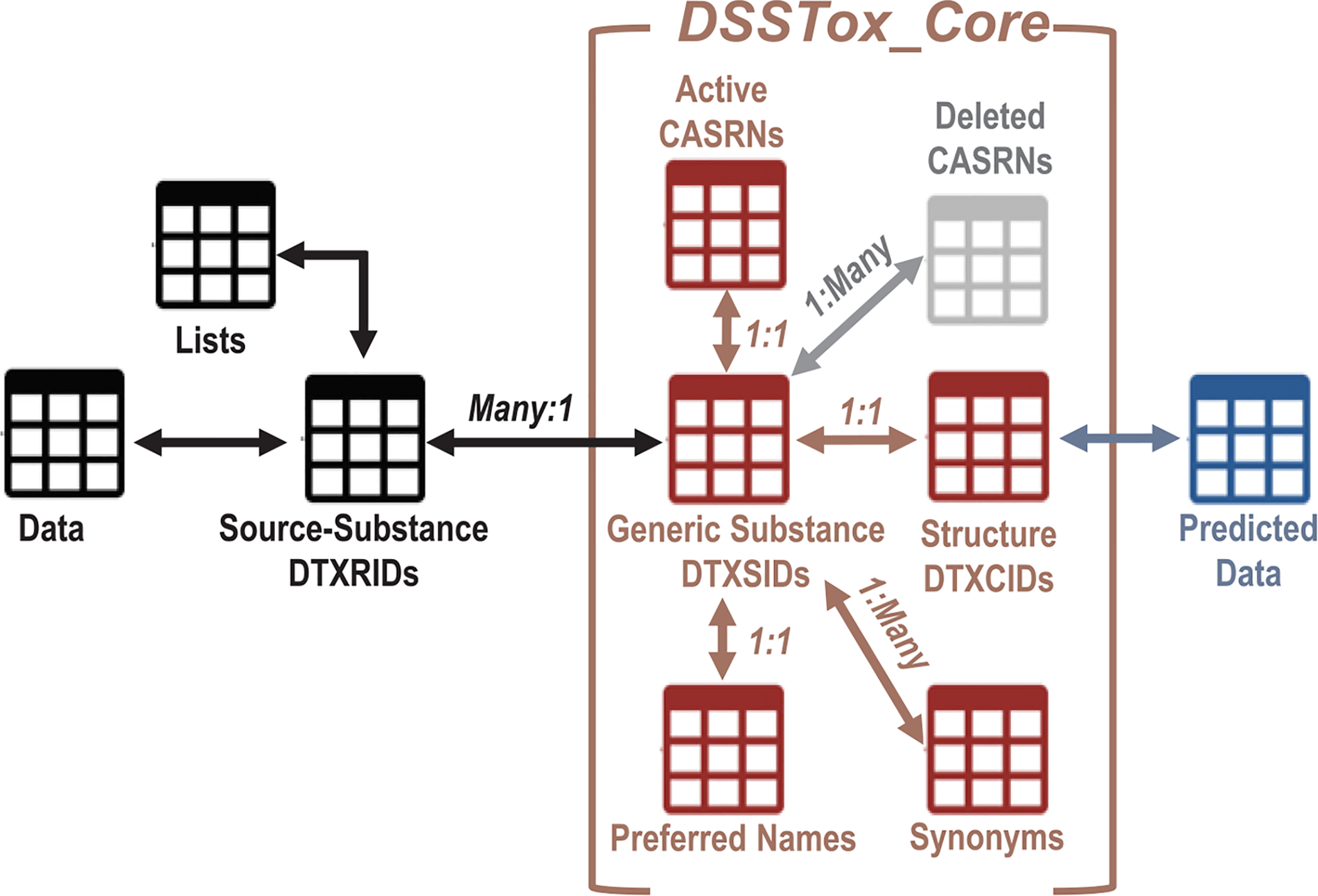
Schematic illustrating the main tabular and relationship components of the DSSTox_V2 data model, centered around the DSSTox_Core substance-structure content.

The above substance-structure identifiers are stored in what we term the “DSSTox_Core” portion of the MySQL DSSTox database. Fig. 1 provides a simplified schematic of the original DSSTox_V2 data model and tables, with DSSTox_Core containing the quality-filtered, curated content of DSSTox that corresponds to what is deemed “truth” for the purposes of mapping and curating new source lists. Importing the original data tables of DSSTox_V1 into this new data model first required implementation of strict mapping rules (indicated as Many:1, 1:1, and 1:Many relationships in Fig. 1), as well as creation of intransient and unique DSSTox-specific IDs suitable for semantic web integration and data exchange. The modified IDs included: 1) a source-substance record ID, **DTXRID**, of the form DTXRID# (i.e., text followed by a numeric index) that is unique to both the source list and the substance (similar to a PubChem SID); 2) a generic substance ID (**DTXSID**), of the form DTXSID#, which is an extension of the original DSSTox_V1 numeric GSID (with no counterpart in PubChem); and 3) a structure ID (**DTXCID**), of the form DTXCID#, which is an extension of the original DSSTox_V1 numeric CID (similar to a PubChem CID).

**Fig. 2.**
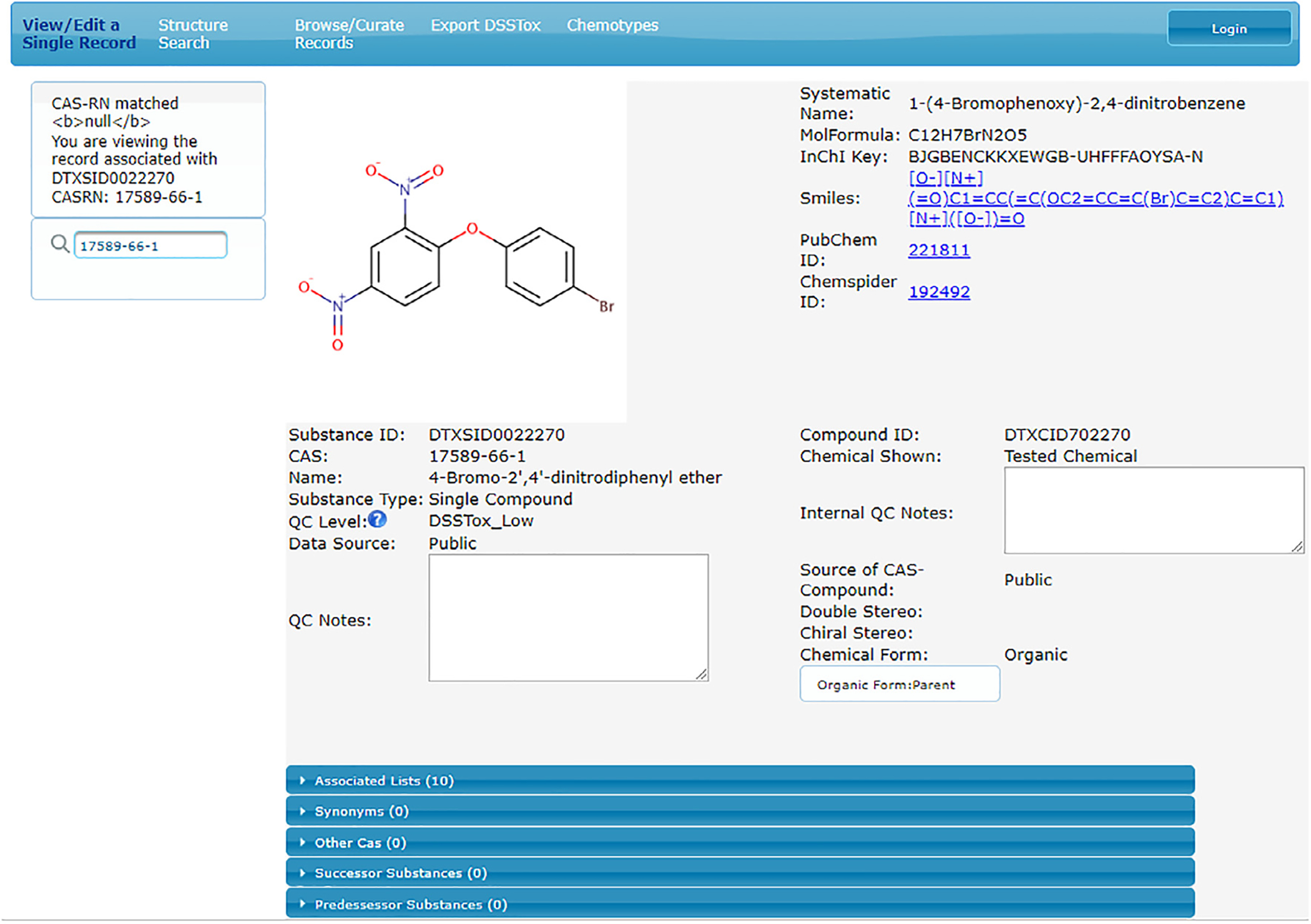
Screen snapshot view of DSSTox ChemReg application, built to provide an interface for trained DSSTox curators to register and edit new and existing DSSTox substance records subject to structure and substance data model controls.

Once the DSSTox_V1 content was migrated to the new DSSTox_V2 MySQL database (*MySQL Community Edition v5.7*), it was necessary to replace what had been manual workflows, which relied on direct curator-editing of primary structure files and spreadsheets of data, with a chemical registration interface to provide DSSTox curators edit privileges and controlled access to the database. Fig. 2 provides a screen snapshot of the ChemReg application interface built for this purpose using Java Server Faces *2.1* with Prime Faces v5.3. The interface contains an embedded ChemAxon MarvinJS v17.26.0 structure-drawing tool, free-text entry boxes (for manually entering CAS RN, Name, and Notes), and several pull-down menu tags.

This interface provides users with a search box where a name, CAS RN, SMILES or ID query will be resolved using the following procedure: 1) retrieve an exact match record; 2) reroute the query through the Deleted CAS RN or synonym table to return a single valid or unique DTXSID record match, identified as such; 3) reroute the query through the synonym table and list two (or more) possible DTXSID record matches (e.g., in the case of a name tagged as “ambiguous”); or 4) return a blank page with the message that the query identifier was not found, providing the curator with the option to begin registering a new substance. A new substance registration proceeds with curator entry of a Preferred Name, a structure (if applicable), and a CAS RN (if available). Optional inputs are record notes, synonyms, and substance relationships. The new substance registration is not accepted by the interface unless two conditions are met: 1) the CAS RN, Preferred Name and structure are not found in the database; and 2) all required menu tags have been selected. If one or more of the three main identifiers (CAS RN, Name, structure) in a new substance registration are located in the database linked to other identifiers (e.g., a newly entered CAS RN is identified as a Deleted CAS RN for a previously registered DTXSID record, or the structure matches another DTXSID record), this creates a mapping conflict that must be resolved by the curator prior to the new registration being accepted. If the new registration is accepted, the record is saved, and a new DTXSID and DTXCID are generated.

**Fig. 3.**
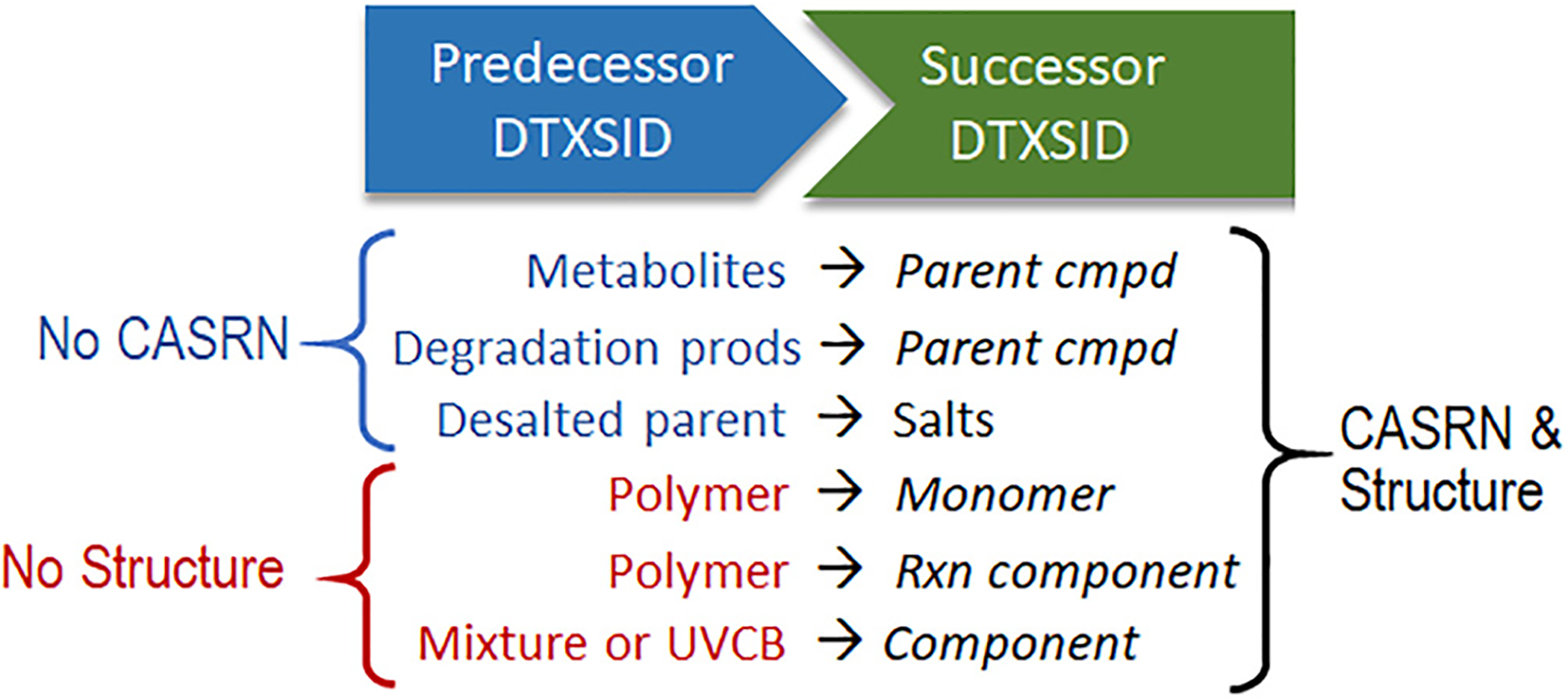
Most commonly encountered cases of “Predecessor” substance records, with either no CAS RN or no structure, mapped to a corresponding “Successor” substance containing a CAS RN and structure.

To facilitate structure-location of data records in online searches, DSSTox_V1 originally allowed for the assignment of a “representative structure” to a non-structurable record, such as for a defined mixture. This created a Many:1 mapping of substances to structure that violated the unique mapping rule. Hence, each DTXSID substance record previously assigned a “representative structure” in DSSTox_V1 was stripped of its structure, and a manually annotated “relationship” linkage of that no-structure record to another DTXSID record, uniquely corresponding to the removed representative structure, was created (in some cases requiring new registration of the structure-substance record). Similarly, given the central role of CAS RN in enforcing the unique substance-structure relationships and guiding curation, when a new substance is registered without a CAS RN, as in the case of many newly identified metabolites and transformation products identified in EPA research projects, a linkage may be created to a closely related DTXSID record that has a CAS RN. In this way, manually curated records in DSSTox_V2 can be mapped to a structure and CAS RN either directly or indirectly through relationship mappings. Several of the most commonly annotated relationship types, in which either a CAS RN or structure is missing in the original DTXSID record, are listed in Fig. 3. Note that these relationship mappings can be applied more generally to any pair of DSSTox records, regardless of whether a CAS RN or structure is missing (see Suppl. Material, Examples 5 and 6 showing how relationships are viewable in the Dashboard).

## 4. DSSTox_V2 expansion & quality curation levels

The original DSSTox_V1 content that was migrated to the DSSTox_V2 data model had undergone extensive manual curation review using both public and commercial resources. Hence, this high-value content provided the first iteration of DSSTox_Core and served as the initial benchmark against which new database inputs were to be judged. Moving forward, these substances were assigned to one of two DSSTox quality curation levels (*qc_levels)* reflecting the degree of curator confidence in the consistency and accuracy of the CAS RN-Name-Structure assignments: 1) *DSSTox_High* was applied to the small minority of records in which the definitive CAS database had to be consulted to resolve conflicts and confirm the CAS RN-Name-Structure associations observed in the public domain; and 2) *DSSTox_Low* was applied to all other records that were reviewed by an expert DSSTox curator using only public information resources, in which a consensus of sources supported the unique CAS RN-Name-Structure assignment.

For the DSSTox_V2 expansion carried out in 2014, portions of three publicly available chemical databases that were being widely used by environmental scientists were sequentially, algorithmically checked for consistency and auto-loaded: the US EPA’s Substance Registry Services (EPA SRS) database [https://iaspub.epa.gov/sor_internet/registry/substreg/] (accessed June 2013; current download access at [http://www.exchangenetwork.net/data-exchange/srs/]); the National Library of Medicine’s (NLM) ChemIDplus (ChemID) database [https://chem.nlm.nih.gov/chemidplus/] (accessed November 2013 through NLM license agreement; current download access at [https://www.nlm.nih.gov/databases/download/chemidplus.html]); and the subset of PubChem compounds associated with one or more CAS RN-type synonyms at the time of download (Unfiltered-Synonyms file accessed September 2014, using PUG system to download structures; current download access at [ftp://ftp.ncbi.nlm.nih.gov/pubchem/Compound/]). The downloaded EPA SRS database was a high-quality public resource of approximately 77 K substances with SRS Registry names, CAS RN and CAS-systematic names (provided by CAS), as well as legacy SMILES added previously by EPA researchers. EPA SRS listed all chemicals covered under EPA’s Toxic Substances Control Act [https://www.epa.gov/laws-regulations/summary-toxic-substances-controlact], as well as all chemicals listed in public EPA regulatory documents. The ChemID database of chemicals, obtained by license agreement with NLM, was a semi-curated collection of approximately 370 K chemicals largely focused on environmental and public health, whose contents had undergone varying degrees of manual review but were primarily compiled from public resources. ChemID provided a rich source of CAS RN, chemical names and structures, but did not enforce uniqueness on either CAS RN or structure in relation to substance. Finally, PubChem provided the largest collection of mol file structures and synonyms (both CAS RN and names) but lacked manual curation review or CAS database verification for its content and, similar to ChemID (whose content PubChem had almost fully incorporated), allowed 1:Many mappings of structure to CAS RN and name. By restricting import of PubChem content to approximately 990 K substances associated with structures and one or more CAS RN-type synonyms, a large number of substances with associated bioactivity data were included and structure-only datasets that served as virtual libraries for drug screening (e.g., ZINC screening library [http://zinc.docking.org/] [29]) were excluded.

**Fig. 4.**
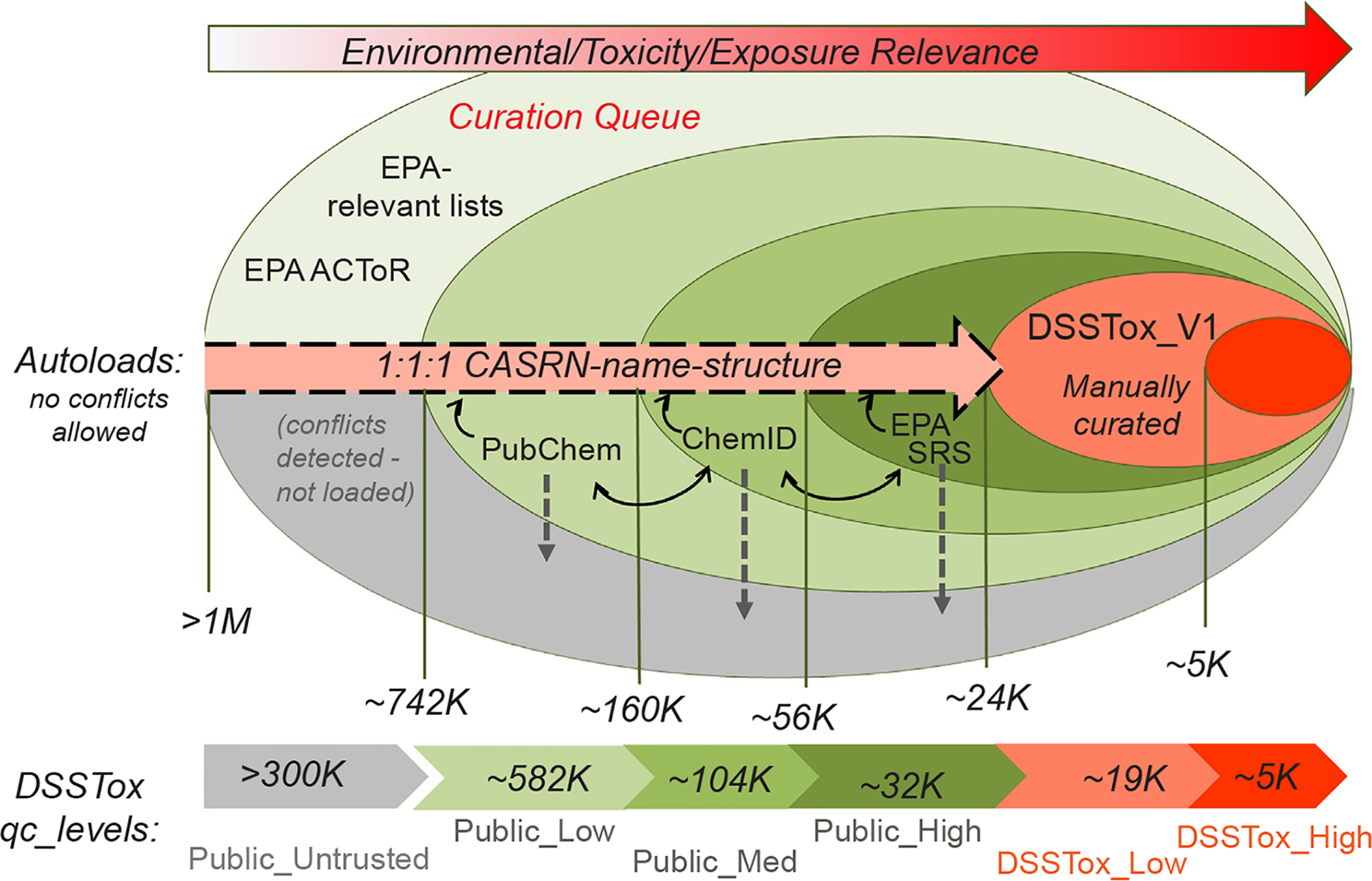
The process by which content from 3 public databases (EPA’s Substance Registry Services - SRS, NLM’s ChemID, and PubChem) was quality filtered, and either assigned to one of five *qc_levels* and sequentially loaded into the DSSTox_Core portion of the DSSTox_V2 data model in 2014 or rejected and placed in the Public_Untrusted bin, requiring further curation review along with other queued EPA lists.

Fig. 4 illustrates the process by which new substance-structure content from each of the three publicly available databases was quality-filtered and sequentially loaded into an expanding DSSTox_Core, significantly building on the original DSSTox_V1 content. The autoload order and *qc_levels* assigned to each public database were based on the historical level of DSSTox curator trust in the identifier associations in each database. Hence, EPA SRS content was loaded first and substances that passed through the consistency quality filters were assigned to the “Public_High” *qc_level*. ChemID content was loaded next, with internally consistent, non-overlapping substances that agreed with PubChem content assigned to the “Public_Medium” *qc_level*. Finally, non-overlapping PubChem content without internal conflicts in identifiers was assigned to the “Public_Low” *qc_level*.

The general process for loading each of the databases involved three steps. First, internal consistency of identifiers within the database was evaluated and substances having identifier conflicts within the database were set aside and not loaded. Next, each database was compared to the “next-level” public database (i.e., EPA SRS content was compared to ChemID, and ChemID content was compared to PubChem) with all conflicts again set aside. (Note that PubChem being the largest and last database to be loaded did not have a “next-level” database for comparison.) After completing these first two steps, the set of substances remaining represented the “cleaned” set ready for loading into each *qc-level*. The portions of these “cleaned” sets that overlapped the growing content in DSSTox_Core were used to quantify the extent of possible mapping errors associated with a *qc-level* while the non-overlapping portion, which could not be further evaluated, was loaded without further checks.

**Fig. 5.**
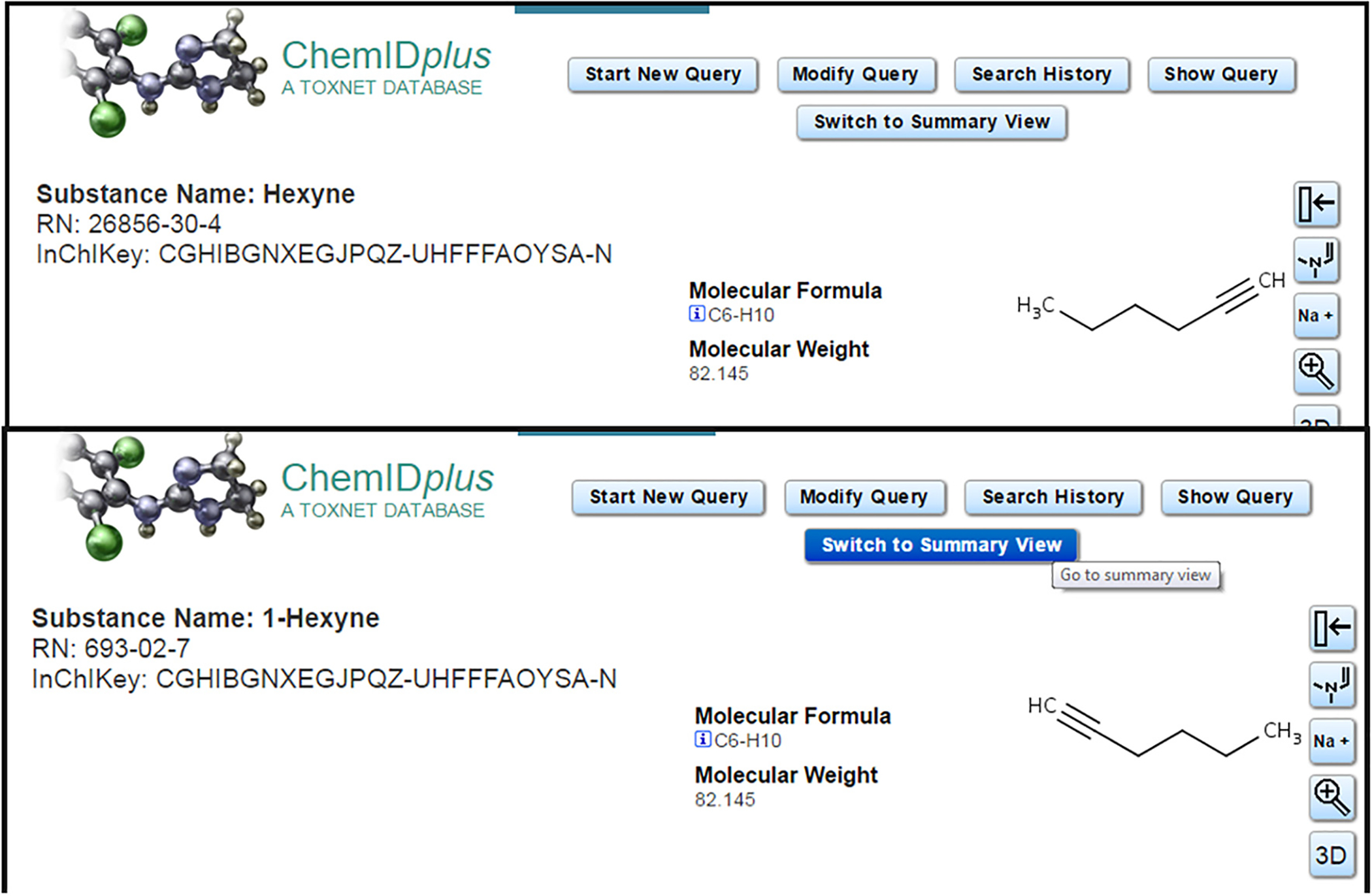
Two ChemID substance records listing the same structure (and InChIKey) for two different Substance Names and CAS RNs. In this case, the Names and CAS RNs are correctly paired, but the structure assigned to the top record is an approximate representation given that the position of the triple bond is unspecified.

Once again, the key constraint applied to this process was the requirement of a unique 1:1:1 mapping of CAS RN-Name-structure for each substance added to DSSTox_Core. Whenever this rule was violated and conflicts were detected, the substance was placed into a quarantined “Public_Untrusted” *qc_level* bin requiring further curation review. An example of two internally conflicting records within ChemID, in which the same structure is mapped to two different CAS RN-Name records, is provided in Fig. 5; in DSSTox, the top substance is currently registered with a relationship (component of mixture) added that creates a linkage to the bottom record to resolve the conflict (alternatively, a Markush structure could be added for the top record indicating uncertainty in the location of the triple bond).

Fig. 4 also indicates the number of records that were successfully added to DSSTox_Core in each sequential autoload step, along with the corresponding *qc_levels* of the newly added content. In this way, DSSTox_Core was expanded from the original 24 K DSSTox_V1 records to more than 740 K registered substances. Unlike the original content of the three public databases, however, each of these newly registered substances satisfied the unique 1:1:1 CAS RN-Name-structure mapping rule within DSSTox and each was assigned to one of five *qc_levels* (DSSTox_High, DSSTox_Low, Public_High, Public_Medium, Public_Low) conveying the degree of curator confidence in the three identifier associations.

## 5. How bad was the problem?

**Fig. 6.**
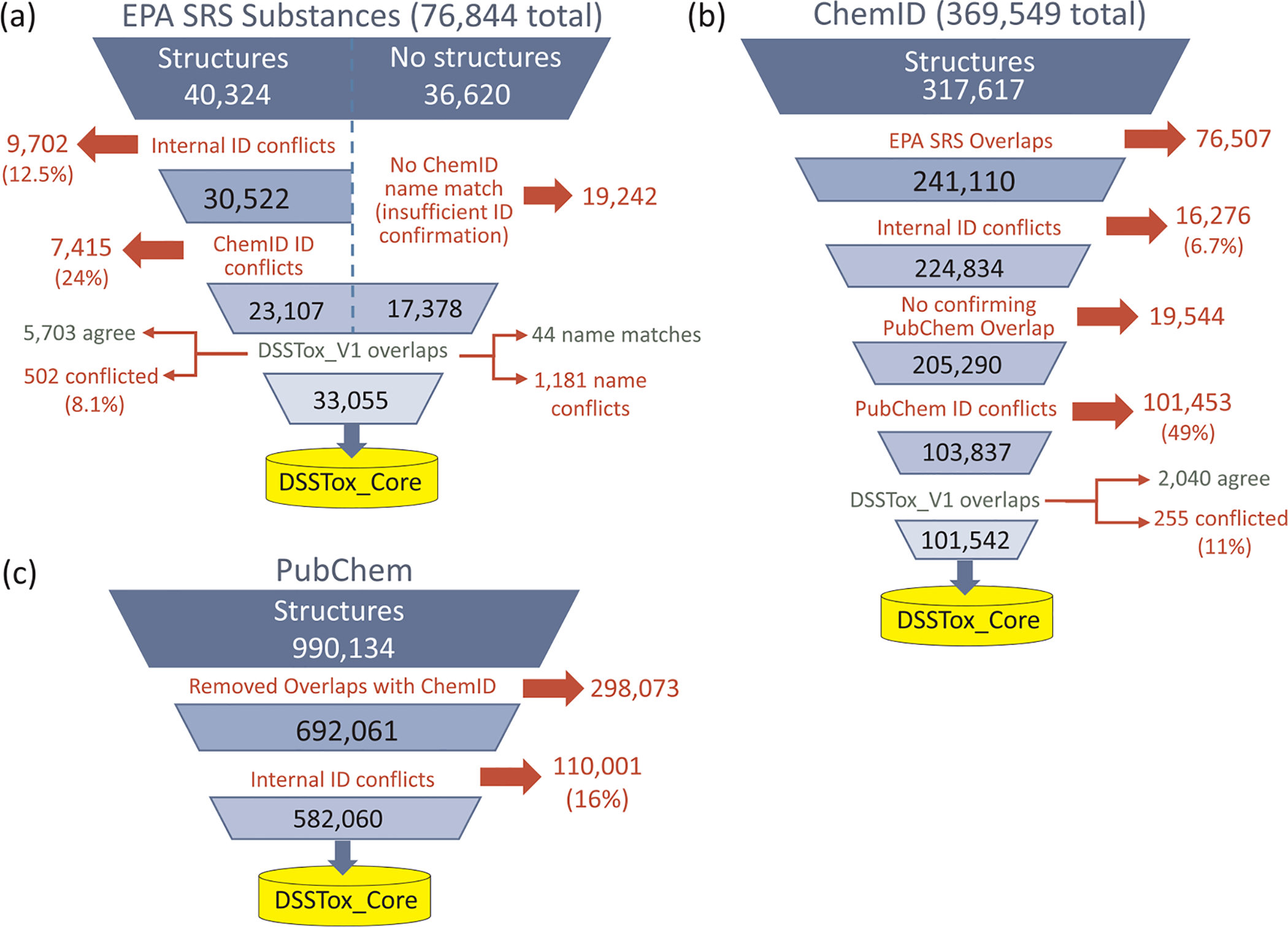
Shown for each of the three public databases that were sequentially added during the DSSTox_V2 expansion phase - (a) EPA SRS, (b) ChemID, and (c) PubChem - is the process by which chemicals were quality filtered, and the numbers of chemicals at each step that were either removed from further consideration or moved forward for possible incorporation into the expanding DSSTox_Core.

Essential to the process of expanding DSSTox_V2 content was the rejection of significant portions of each of the three public databases that violated the 1:1:1 CAS RN-Name-structure rule, either internally (i.e., conflicts in identifiers across substances within a database as in Fig. 5), or by comparing substance IDs to those in the next-level public database. The rejected content from the three public databases, in turn, revealed for the first time the full extent of mapping inconsistencies associated with these public databases. Fig. 6(a)–(c) provide a more detailed view of the process by which each of the three public databases was evaluated and filtered prior to adding new content to DSSTox_Core, indicating the numbers and percentages of conflicting content detected at each filtering step.

The percentages of internal conflicts detected in portions of the three databases, primarily based on CAS RN-InChIKey inconsistency (but also including CAS RN-Name inconsistencies for unstructured EPA SRS content), ranged from 6.7% in ChemID to 16% in the portion of PubChem not overlapping with ChemID.^7^ The relatively high identifier conflict rate within EPA SRS (12.5%) was attributed to the lack of definitive structural content in EPA SRS; SMILES, IUPAC names, and index names (converted to structure using an implementation of OPSIN, [https://opsin.ch.cam.ac.uk/]) were all used to create InChIKeys for a single record and, when those InChIKeys did not agree, the record was flagged and excluded from the load. In addition, EPA SRS had representative SMILES added to many mixture and UVCB records. Conflicts in overlapping content between databases was 24% in the overlap of internally consistent EPA SRS records with ChemID (out of 30,522 structure-containing records) and 49% in the overlap of internally consistent ChemID records (minus EPA SRS) with PubChem (out of 205,290 total records). It must be noted that inconsistency checks on PubChem were carried out using unfiltered synonym lists out of an abundance of caution, which increased the likelihood of finding conflicts. Once internal and cross-database checks were completed, the “cleaned” portions were compared to the overlapping portion of DSSTox-Core’s manually reviewed content, indicating an 8.1%, 11% and 16% inconsistency rate in the Public_High, Public_Medium, and Public_Low *qc_levels* content, respectively. This is a reasonable estimate of CAS RN-structure mapping error rates within these *qc-levels*; however, since there is undoubtedly some error in the manually curated DSSTox-Core content (the majority of which is DSSTox_Low) and the sample sizes for this comparison were a fraction of the total databases, error rates in the full content of each *qc-level* could vary. The high percentages of internal and cross-database conflicts are largely attributed to the Many:1 relationships of CAS RN and names to structure, as well as to the common practice of adding representative structures to substances that cannot be accurately documented with a single defined structure (see Suppl. Material, Example 6).

It should be noted that the numbers in Figs. 4 and 6 are the result of a singular process that took place in 2014 using a downloaded snapshot version of each of the three public databases available at the time. Each of these databases has undergone growth and modification since then, particularly significant in the case of PubChem, but none has adopted the strict 1:1:1 CAS RN-Name-structure mapping rule supported by manual curation to resolve conflicts; hence, although the totals would undoubtedly change if this exercise were repeated today, a large number of identifier conflicts would likely persist. This exercise if repeated today would also be confounded by the fact that a recent version of the publicly available, curated DSSTox content has been deposited into PubChem. Hence, whereas the level of conflicts with DSSTox content will undoubtedly be reduced, the unmatched and uncurated non-DSSTox overlapping content will likely maintain similar levels of inaccuracy as generally found in the public domain.

It should also be noted that the expansion phase of DSSTox_V2 succeeded in achieving two goals: 1) a 34-fold increase in DSSTox_Core content, providing greatly improved coverage of the environmental chemical landscape; and 2) preservation of the 1:1:1 CAS RN-Name-structure mapping rule across all DSSTox_V2 registered content and the addition of quality metrics to convey curator confidence in identifier associations for the auto-loaded content. The *qc_levels*, in turn, are helping to guide current curation efforts towards improved accuracy (e.g., by upgrading a Public_Low record to DSSTox_Low or DSSTox_High upon further review). Thus, although the 1:1:1 rule does not provide a guarantee that DSSTox_V2 content is 100% accurate, when the rule is violated inaccuracy is virtually guaranteed.

Finally, given the environmental relevance of EPA ACToR content, it was of interest at the time of the DSSTox_V2 expansion to determine how much of ACToR’s CAS RN content was captured after significant structure-containing portions of EPA SRS, ChemID, and PubChem were autoloaded into DSSTox_Core. At the time, ACToR contained approximately 560 K CAS RN-ID’d records and approximately 300 K of those CAS RNs, more than half, were determined to be missing from DSSTox_V2. Of those missing, it was found that approximately half (150 K) had one or more conflicted IDs (name or structure) in comparison to DSSTox_Core content. Whereas a large portion (100 K) of this initially unregistered ACToR content was recently loaded using a similar automated approach to that in Fig. 4, the remainder, combined with the rejected, conflicted content from each of the three auto-loaded public databases, represents a large, unresolved curation backlog for the DSSTox project that remains a challenge to limited DSSTox curation resources.

## 6. Post expansion: The DSSTox curation challenge

Since the initial expansion of DSSTox_V2 to 740 K records in 2014, tens-of-thousands of new substances have been registered using a combination of automated and manual curation processes. Typically, chemicals enter the queue for curation from lists submitted by EPA researchers and collaborators. Lists are prioritized based on relevance and value to EPA regulatory and research programs and to the environmental research community at-large. Each list, in turn, consists of “source substances” represented by a set of source identifiers. Generally, a single source substance will have at least one chemical name identifier, often with an associated CAS RN, but only rarely is it accompanied by a structure. DSSTox list curation today bears many similarities to the processes outlined above, in which the three public databases were auto-loaded. DSSTox list registration generally proceeds in three stages: 1) initial auto-mapping of source content to existing DSSTox_Core content; 2) identification and binning of identifier conflicts according to type; and 3) manual curation review and resolution of all conflicts. Partial list registration can proceed automatically, with source substances auto-mapped to the algorithmically defined “best” generic substance, whereas full list registration almost always requires some level of conflict resolution through manual curation and often requires registration of new substances. In addition, when curating new lists, Public *qc_level* records may be subject to further manual review and can be edited and elevated to DSSTox_Low (if there is sufficient consensus of public resources) or DSSTox_High (if confirmed in the CAS database).

**Fig. 7.**
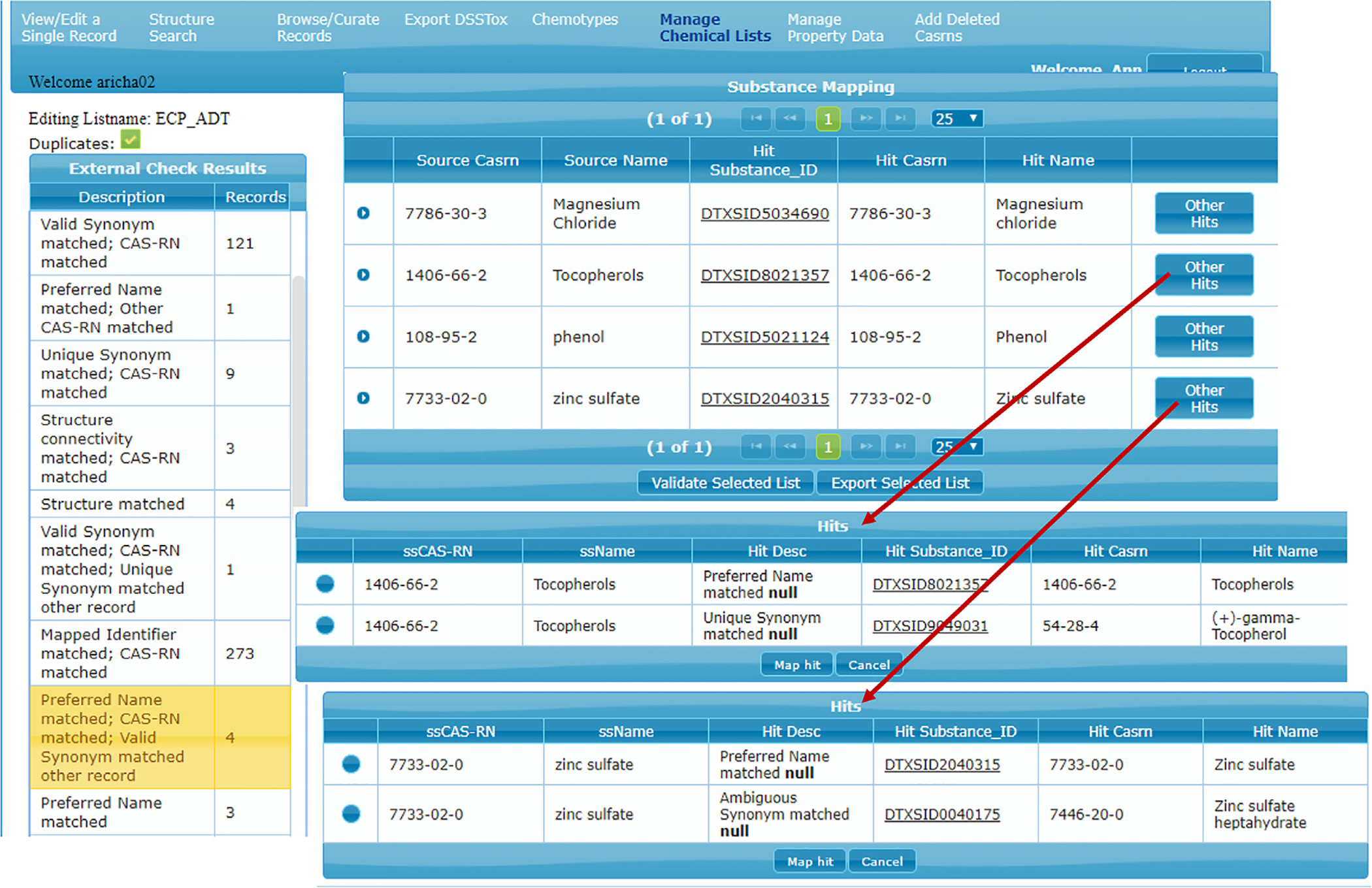
Snapshot view of the DSSTox Curation Interface used by DSSTox curators to register lists; shown on the left are the totals in the various identifier conflict bins that remain to be curator-validated, where each bin and each conflicted record within each bin can be accessed by the curator (2 expanded views shown).

A sample screen shot of the DSSTox list curation interface supporting the above curation workflow is provided in Fig. 7, with several possible types of ID conflicts listed in the left panel. Each type of conflict presents different challenges to the curator. The panel at the bottom of Fig. 7 provides an example where the source CAS RN maps to one DTXSID and the source name maps to another DTXSID.

An area where DSSTox curation has proven essential is in support for EPA’s ToxCast testing program, where accurate identification and DSSTox registration of physical samples submitted for testing is integral to the internal ToxCast Chemical Management system (ChemTrack). ChemTrack was developed within EPA to track all ToxCast samples from procurement through to plating and shipment, and to provide definitive chemical mappings in association with all ToxCast assay data generated from plated samples. Over the past decade, ToxCast has screened upwards of 4500 distinct substances in a wide range of HTS assays, generating millions of assay-data points that provide a rich source of data for predictive modeling. Almost all ToxCast substances are procured from commercial sources and DSSTox chemical registration relies upon the veracity and completeness of documentation provided by those commercial sources. As in other areas of DSSTox curation, it was realized from the earliest days of the ToxCast program that chemical supplier-provided information was as fraught with identifier conflicts and errors as other types of public chemical information. It was previously reported that nearly 4000 supplier-provided structures for the ToxCast chemical library (version dated January 2016), when compared to the final DSSTox registered structures, had an error rate (i.e., different InChIKeys) of 22%, with half of the errors (11%) resolved on desalting largely attributed to missing salt and hydrate information, 8% of the errors resolved at the molecular formula level attributed to missing stereochemistry or geometric isomers, and the remaining 3% gross errors of a more serious nature [15]. As a result, a workflow was instituted early on in which Certificates of Analysis (CoAs) and Material Safety Data Sheets (MSDSs or SDSs) were required of suppliers, whenever possible, and were consulted during curation review since these documents (particularly CoAs, when available) were found to provide the most reliable name and CAS RN information. Supplier-provided structures were found to be sufficiently unreliable that they are not used by DSSTox curators when registering samples. DSSTox curation has also provided substance registration support for the Tox21 program since its inception, but the additional chemical sample curation review is only provided for the EPA-controlled sample portion of Tox21 (approximately a third of the total), with the remaining portions of Tox21, contributed by NTP and NCATS, undergoing DSSTox curation as source lists in the usual fashion.

## 7. Where are we and where are we going?

**Fig. 8.**
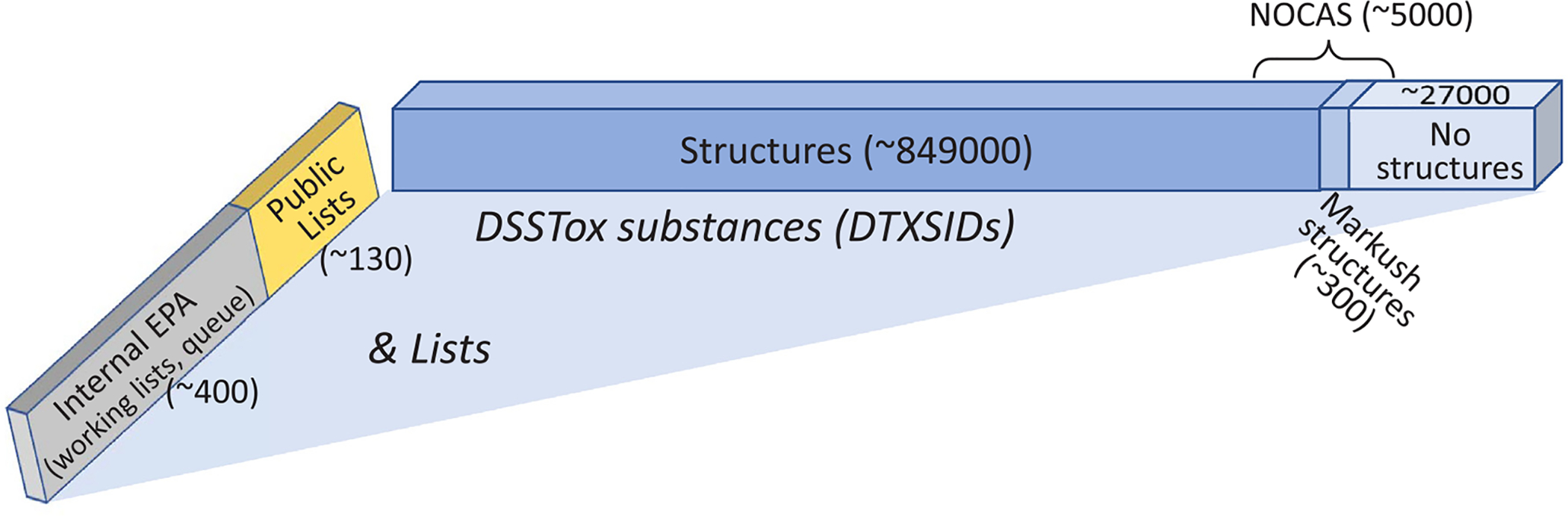
Total numbers of DSSTox substances and registered lists (public and Internal EPA) as of February 2019.

A snapshot of DSSTox_V2 content totals (as of February 2019) is presented in Fig. 8. These include the number of substances with or without structures or CAS RN (including mixtures and UVCBs), the number of registered category substances (including manually mapped and Markush structures, *vide infra*), and the numbers of public and non-public registered DSSTox lists.

Less visible within the Dashboard, but stored and used within DSSTox for search redirects, are synonyms and source names, which number in the millions, and Deleted CAS RNs, associated with approximately 35% of the DSSTox_High substances and numbering in the tens-of-thousands. Whereas synonyms and Deleted CAS RNs (which can be considered a type of synonym) are exceedingly useful for locating all data records associated with a substance, they create challenging mapping issues if not condensed to the appropriate, uniquely mapped substance. The full extent of the problem is illustrated by a substance named “Bisphenol A/Epichlorohydrin resin” (DTXSID0050479 [https://comptox.epa.gov/dashboard/DTXSID0050479]) registered with Active CAS RN 25068-38-6, which has 664 synonyms stored in DSSTox, of which 316 are Deleted CAS RNs listed in the CAS database.

In addition to its quality-filtered content and supporting data model, DSSTox is distinguished by the focus of its manual curation efforts on chemical lists of greatest interest and importance to the environmental research and regulatory community. Examples within the Dashboard include: EPA’s Hydraulic Fracturing list, EPAPHF, containing more than 1200 substances, publicly available at [https://comptox.epa.gov/dashboard/chemical_lists/epahfr]; EPA’s Toxic Substances Control Act (TSCA) inventory, with more than 35 K substances curated thus far, internal EPA-only, with a public version of the non-confidential inventory available at [https://comptox.epa.gov/dashboard/chemical_lists/tscaactivenonconf]; EPA’s Consumer Product Database (CPDat) [30], containing more than 40 K substances, with more than 30 K publicly available at [https://comptox.epa.gov/dashboard/chemical_lists/cpdatlist]; and, most recently, PFASMASTER, a list of more than 5000 perfluorinated alkyl substances (PFAS), publicly available at [https://comptox.epa.gov/dashboard/chemical_lists/pfasmaster], the majority of which were first published with CAS RN (and without structures) in the Organisation for Economic Co-operation and Development (OECD) Global PFAS database [http://www.oecd.org/chemicalsafety/portal-perfluorinated-chemicals/], publicly released in March 2018. Each of these lists presented unique challenges to DSSTox curators, and, as DSSTox-registered lists, each has significantly enriched the public reservoir of quality substance-structure information. In the case of EPA’s Hydraulic fracturing list, the challenge was in reconciling many sources of aggregated public chemical names and CAS RN containing numerous identifier errors and conflicts [31]. In the case of CPDat, DSSTox curators are often presented with chemical names only, with lack of chemical specificity and frequent errors. Similar challenges in dealing with the inconsistencies and ambiguity of chemical names have been most recently reported in reconciling metabolite names in biochemical databases used for genome-scale metabolic modelling [32]. In the case of the PFAS inventory, curators faced challenges in assigning preferred names that conformed to published naming conventions and notions of categories (e.g., perfluorosulfonic acids, commonly abbreviated PFSA), in assigning structures to structurable substances whose names could not be resolved with available name-to-structure tools, and in shortening extremely long systematic names for a significant number of structurable chemicals, polymers and esters.

More generally, DSSTox, both internally and as accessed through the public Dashboard, is distinguished from most other public databases by its robust list-handling functions. Lists not only provide the primary means for seeding new chemicals and chemical-list associations into DSSTox, but list curation and validation of source ID mappings goes far beyond most other public efforts and utilizes all source IDs, not just structures (see Fig. 7). List registration serves to maintain and convey the relationship of a set of chemical substances to a particular data source (e.g., data from DrugBank [https://www.drugbank.ca/]). In addition, lists are a major organizing principle for data exploration and delivery within the Dashboard [18]. Any public DSSTox list (132, as of April 15, 2019) can be accessed from the “Lists” tab on the main Dashboard menu bar [https://comptox.epa.gov/dashboard/chemical_lists], and can be viewed, sorted, filtered and exported in multiple formats. A user can also choose to “Send” the list to the “Batch Search” page, where it can additionally be populated with metadata and precomputed intrinsic or predicted properties or cross-referenced to one or more other published DSSTox lists (to determine overlapping content). These easy-to-use Dashboard capabilities are greatly increasing the speed and efficiency of data gathering efforts within and outside of EPA and are preventing errors that are easily introduced when these types of tasks are carried out manually (particularly by non-chemists). Finally, lists are being used to underpin capabilities of other customized Dashboard views, such as for EDSP21 ([https://www.epa.gov/endocrine-disruption/endocrine-disruptor-screening-program-edsp-21st-century], [https://comptox.epa.gov/dashboard/chemical_lists/edspuoc]) and ToxCast ([https://www.epa.gov/chemical-research/toxicity-forecasting], [https://comptox.epa.gov/dashboard/chemical_lists/toxcast]).

Two chemical database efforts that are of particular note within the environmental regulatory and research field are the commercial CAS product, CHEMLIST® (Regulated Chemicals Listing) [https://www.cas.org/support/documentation/regulated-chemicals] and the chemical content accessible from within the publicly available OECD QSAR Toolbox [http://www.oecd.org/chemicalsafety/risk-assessment/oecd-qsar-toolbox.htm]. CHEMLIST is a searchable database product from CAS that contains more than 150 lists of regulated chemicals in key markets worldwide, spanning 348,000 substances. As is the case with the larger CAS database, this resource is neither publicly available nor can the content be downloaded or combined with other public databases. However, since versions of the chemical lists included in CHEMLIST are publicly available, some are already registered in DSSTox (e.g., TSCA), and any of the remaining lists could be curated and registered to provide structure-annotated lists to the community in a free and open platform. The OECD QSAR Toolbox [33] is a publicly downloadable application that supports user-guided chemical profiling and read-across analysis. Toolbox functions are powered by several underlying chemical structure lists, including versions of EPA’s TSCA inventory and DSSTox, that can be exported from within the application. Given the need for accurate chemical structures to support the functionality of the Toolbox, developers faced many of the same challenges as DSSTox in reconciling conflicting public sources of information. They reportedly employed automated tools to bin and assign quality ratings, similar to those adopted in DSSTox, to reflect confidence in the relationships found among CAS RN, name and structure IDs: High - trustworthy source, Moderate - concurrence of 3 or more public sources, Low - concurrence in 1 or 2 public sources, Conflict - CAS RN from equally reliable sources maps to multiple structures; and N/A - quality can’t be determined due to missing information. These quality ratings in association with structures are conveyed to users; however, there is no indication that a manual curation effort of the sort implemented in DSSTox was employed to resolve conflicts and improve the quality of the substance-structure content.

Several areas of chemistry that are underdeveloped in DSSTox, as well as in other public chemical databases, pertain to inorganics, polymers, mixtures, and UVCBs. Inorganics and polymers represent significant domains of chemical study but are usually excluded entirely from QSAR studies, which typically focus only on organics. In the case of inorganics, DSSTox and other public databases, such as PubChem and ChemID, attempt to render a structure that includes all atoms in a fixed stoichiometric ratio, and to correctly represent the valence of the coordinated metal(s). Whereas drawing guidelines for inorganics exist [34], they do not provide a standardized, agreed-upon method of structure-rendering of coordinated bonding patterns with metals for digital databases (see, e.g., [http://www.chemspider.com/Search.aspx?q=ferrocene]); hence, the structure serves primarily to structure-locate the metal-containing record in searches. In the case of polymers, no DSSTox structure is assigned, but DSSTox curators can manually add a relationship linkage to a precursor monomer or reaction starting material, if known or provided by the source (Fig. 3). However, pertinent details to a polymer chemist (and toxicologist), such as average polymer length, nature and size of repeating units, reactive functional groups, cross-linkages, starting materials and their ratios, etc., are typically not captured.

**Fig. 9.**
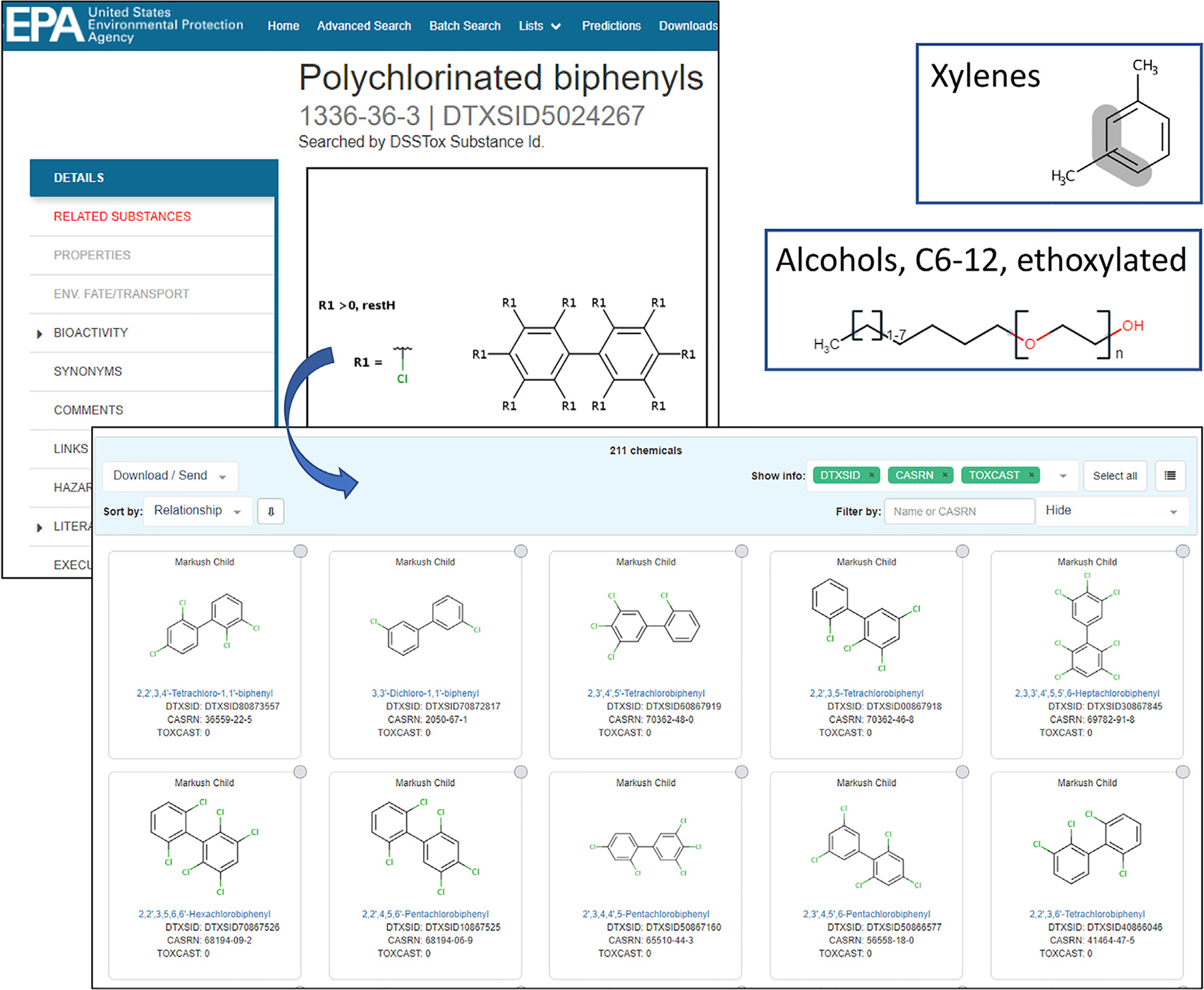
Dashboard view of three types of Markush structures, with a representative sample of the 100 enumerated “child” structures shown for “Polychlorinated biphenyls”, as retrieved under the tab “RELATED SUBSTANCES” [https://comptox.epa.gov/dashboard/dsstoxdb/results?search=DTXSID5024267#related-substances].

The inability to assign a unique, defined structure to polymers, mixtures, and UVCBs is particularly problematic in the environmental field, where large numbers of these substances are listed in regulatory documents. In the case of EPA’s TSCA inventory, almost 40% of the listed substances cannot be mapped directly to a single defined structure. In addition, regulations often consider categories of chemicals that are loosely defined textually (i.e., by name fragments) rather than with clear structural rules and boundaries (e.g., PFAS, triazines, conazoles). Several mechanisms are available for capturing concepts relating to chemical categories or chemical groupings within DSSTox. The first employs list registration, in which series of related chemicals are grouped by structure, use-category or function. Currently published lists of this type in the Dashboard include hazardous algal bloom chemicals (ALGALTOX [https://comptox.epa.gov/dashboard/chemical_lists/algaltox]) and Bisphenols (BISPHENOLS [https://comptox.epa.gov/dashboard/chemical_lists/BISPHENOLS]). A second means for creating categories is through use of manually added relationship tags (as in Fig. 3). Until recently, the category “Polychlorinated biphenyls” or “PCBs” was populated in this manner [https://comptox.epa.gov/dashboard/DTXSID5024267]. Since the PCB category is strictly bounded to 209 potential isomers, 209 linkages were manually added to allow for a full retrieval of all possible isomer structures in the Dashboard [https://comptox.epa.gov/dashboard/dsstoxdb/results?search=DTXSID5024267#related-substances]. Finally, a third means of creating categories utilizes recently implemented ChemAxon structure-drawing capabilities that support Markush structures. Markush structures allow for specification of varying chain lengths, repeating units, R-group chemistry, substitution patterns, and query conditions to be placed on the category. Shown in Fig. 9 are three sample Markush structures, with a representative sample of the 209 linked substances, or “children” that were automatically identified based on the Markush structure shown for “Polychlorinated biphenyls” (which has an assigned CAS RN).

Each of the above means for representing and registering categories within DSSTox is manually intensive and requires significant curator expertise. Markush structure-processing technology, however, provides the only currently available means by which enumeration of “child” structures from a single “parent” Markush structure can be automated. DSSTox curators are registering Markush structures for a limited number of categories within high-priority EPA lists, at present. As these become more widely accepted, understood, and used, it will help to enforce a greater degree of structural clarity and consistency in the use of category terms within the environmental research and regulatory communities.

Another area of development in delivering DSSTox content to the public is in providing structure, substructure and similarity searching through the public Dashboard.^8^ Structure-based searching is of value when a chemical name or CAS RN is not in the database, enabling a user to locate an exact or highly similar structure matching record. For example, a user might have a newly synthesized chemical or newly identified metabolite and is interested to find either an exact match or the most similar ToxCast chemical with associated HTS data. Substructure searching allows a user to retrieve all chemicals (and associated data) containing a particular structural fragment or scaffold, creating a user-defined category. Finally, similarity searching is used to identify data-rich analogs to the query structure, which in turn can provide a starting point for a QSAR or read-across approach. For any DSSTox substance mapped to a structure, the current Dashboard allows for viewing of “Similar Compounds” from a precomputed listing based on a Tanimoto similarity coefficient with a threshold of 0.08, based on default fingerprints provided in Bingo’s PostgresSQL implementation [https://lifescience.opensource.epam.com/bingo/bingo-postgres.html]. DSSTox’s ChemReg application currently uses the commercial ChemAxon JChem cartridge for structure handling, but in an effort to keep our externally facing applications free from licensing concerns, we have elected not to integrate JChem into the Dashboard. Rather, work is in progress to introduce structure-related searching to the Dashboard using Open Source software (ePam Bingo NoSQL plugin, http://lifescience.opensource.epam.com/bingo/bingo-nosql.html) which requires indexing the content of DSSTox into a NoSQL database. The Ketcher 2.0 JavaScript drawing editor [http://lifescience.opensource.epam.com/ketcher/] is being deployed as the input interface for chemical structure-based queries. These search capabilities will be made available in a future release of the Dashboard via the Advanced Search tab [https://comptox.epa.gov/dashboard/advanced_search/index], which is presently limited to mass and formula searches.

Whereas DSSTox is the container for all chemical substances and substance relationships supporting EPA’s computational toxicology research, other databases are critical components of the overall solution to serve up chemical data of interest to environmental scientists. ChemProp stores both experimental and predicted physicochemical property data of various types. Experimental data have been harvested and curated from online sources such as the PHYSPROP database [28], from EPA databases such as ECOTOX [https://cfpub.epa.gov/ecotox/], and from the peer-reviewed literature. Predicted data are generated using the OPERA models developed by our team [21], by the NICEATM (NTP Interagency Center for the Evaluation of Alternative Toxicological Methods) models [35], by TEST models [https://www.epa.gov/chemical-research/toxicity-estimation-software-tool-test], by EPI Suite and ECOSAR ([https://www.epa.gov/tsca-screening-tools/epi-suitetm-estimation-program-interface], [https://www.epa.gov/tsca-screening-tools/ecological-structure-activity-relationships-ecosar-predictive-model]), and from ACD/Labs prediction models []. Predicted data are based on QSAR and QSPR (Quantitative Structure-Property Relationship) models and are, therefore, available only for chemical substances with chemical structures. Experimental properties, however, can be measured for complex substances such as UVCB chemicals and, thus, may be available.

“QSAR-ready forms” of chemical structures are generated using a KNIME workflow [28] to produce chemical structures that have been desalted and have all stereochemistry and isotopically labeled nuclei removed. These “QSAR-ready” chemical structures are the input files for OPERA prediction models and are mapped inside the DSSTox database with separate DTXCIDs. “MS-ready forms” [36] are related to the QSAR-ready structures except for the deduplication of equivalent moieties in mixtures. For example, in the case of a multicomponent salt with two or more equivalents of a potentially active ingredient, the multicomponent salt as a whole would be removed during QSAR-ready processing. In MS-Ready processing, the chemical would be desalted and the multiple components deduplicated to a single MS-Ready component. The mappings and associated database queries allow for structural neighbors of a substance to be surfaced through the linked substances panel on the chemical details page of the Dashboard.

Finally, one of the primary tenets motivating the development of DSSTox, and the associated Dashboard, is providing access to the underpinning data to allow for community reuse and repurposing. A number of files, generally in either Excel XLS or SDF format that were generated using specific queries against the Dashboard-released version of the DSSTox database are available on the Downloads page on the Dashboard [https://comptox.epa.gov/dashboard/downloads]. The Dashboard links to date-versioned files registered on the EPA Figshare account [http://epa.figshare.com] and includes digital object identifiers (DOI). The list of all available download files as of this writing is provided. Additionally, towards the ever-elusive goal of 100% accuracy of chemical substance-structure content, Dashboard and DSSTox users are encouraged, and a means is provided through the Dashboard, to report suspected or confirmed errors found in DSSTox content via the Submit Comment capability [https://www.epa.gov/chemical-research/comptox-chemistry-dashboard-help].

## 8. Conclusion

In summary, what began almost 20 years ago as a small, manually curated database consisting of a handful of chemical structure data sets of interest to the environmental research community and QSAR researchers has evolved into a database spanning over three-quarters of a million substances that is underpinned by strict quality curation processes and supported by modern cheminformatics tools. The DSSTox project is distinguished both by its focus on the environmental research chemical landscape, as well as by the strategic combination of error-detection ability coupled with manual and automated curation processes to bin and resolve substance identifier conflicts. To this end, the importance of CAS RN as a unique and verifiable identifier for establishing accurate substance-structure-data linkages of historical data in the public domain and providing a baseline “truth” standard for DSSTox curation cannot be overstated. The CAS database, with abstracting and associated data and structure search-retrieval services, currently exceeding 146 million registry records [https://www.cas.org/about/cas-content], has served as an invaluable resource to the chemistry and environmental research communities for over a century [37,38]. However, CAS is ultimately an indexing service linked to proprietary content; it is not the intent of the DSSTox project to create an alternative indexing service, but rather to shift the emphasis to accurate structure-indexing and elevate the coverage and accuracy of structure-data linkages in the environmental research realm. Furthermore, it should be noted that even the “definitive” CAS database is not 100% accurate given that it also deals with public information and employs manual curation for data entry, nor does it provide complete coverage of the chemical landscape of interest to environmental researchers, as evidenced by DSSTox NOCAS records. In several instances where DSSTox curators have reported possible inaccuracies of structure drawings in CAS records, this interaction has led to a correction in the CAS database.^9^ Similarly, there are several known cases where DSSTox curators originally assigned a “NOCAS_##” identifier to a substance that was not yet listed in the CAS database, only to find at a later date that a new CAS record had been created and the “source” of the record listed by CAS was DSSTox.^10^

These formal and informal interactions underscore the inter-connected nature of the world of chemical databases and the benefits of all parties working towards a common goal of improving the accuracy of public chemical information in relation to the environment and public health. Towards this end, DSSTox is continuing efforts to harmonize chemical databases within EPA (most notably with EPA’s SRS database) and data are openly shared and registered with several other public databases (such as UniChem [https://www.ebi.ac.uk/unichem/] and PubChem) leading to its usage in other data aggregation efforts [39]. In this way, curated DSSTox substance-structure content for chemicals of particular relevance to environmental and public health enters the public chemical data sphere, expanding and potentially elevating the accuracy of the content of other databases. It should be noted, however, that the constraints of the DSSTox data model (and the enhanced structure-stereo handling of v3000 SDF format) do not necessarily travel with its data, leading to potential quality degradation of content when accessed outside of the EPA Dashboard (see Suppl. Material, Example 7).

The DSSTox database, both within EPA’s larger database environment and as surfaced through the public Dashboard, has come to play an increasing role in supporting a wide range of EPA programs as list coverage, data linkages and advanced capabilities (such as support for QSAR and NTA research) have expanded. Although the feasibility of integrating large public databases towards an improved accuracy ideal without a large manual curation effort has been called into question (see, e.g., Hersey et al. [40]), we have demonstrated that a tiered approach with limited and strategic application of manual curation resources and limited access to the CAS database, supported by *qc_levels* conveying curation confidence in individual records, can effectively complement proven structure-handling solutions towards achieving this goal. And although much work remains to achieve dynamic coordination and fully harmonized chemical structure content in public databases, much progress towards this goal within EPA has been achieved over the past 15 years. In conclusion, the purpose of this article was to relate the history of the DSSTox project and database development, and the underlying curation processes that provide a working model for elevating the accuracy of the public reservoir of chemical substance-structure content to better serve the needs of the computational toxicology research community.

## Supplementary Material

Supplement1

## Footnotes

1 The original DSSTox website, with over 100 pages of supporting information content, was publicly available until 2015. After an EPA-wide website domain update in 2015, DSSTox website migration was not undertaken in lieu of new DSSTox and dashboard tools in development at the time. However, the original website contents remains available in archived format at https://www.epa.gov/chemical-research/distributed-structure-searchable-toxicity-dsstox-database.

2 Although several other content and application-specific chemical databases have been made publicly accessible, to-date, PubChem and ChemSpider remain two of the largest and most-widely used structure-searchable databases,

3 Note that a true “Alternate CAS RN”, i.e. a replicate CAS RN for the same structure, rarely occurs in the CAS database and, when detected and confirmed by DSSTox curators, is either resolved in DSSTox curation and mapped to two distinct DSSTox substances, or the structures are collapsed through InChI and the Alternate CAS RN is added as a synonym to the most recently issued, Active CAS RN substance.

4 As of 6/15/2019, DSSTox is eliminating use of “NOCAS_#####” identifiers as default place-holders and will simply leave the CAS RN field blank if there is no known CAS RN for the substance; all legacy NOCAS will be stored as “synonyms” to link to historical data.

5 Note that DSSTox curators have encountered examples of what we term “dummy CAS RNs” in the public domain that have the same hyphenated format of a CAS RN and may even satisfy the CAS checksum rule, but that are not found in the CAS database (see, e.g., dummy CAS RN 99241–24–4 listed on multiple chemical supplier websites and as a synonym in PubChem SID 364176756, closely corresponding to (2-Hydroxypropyl)-alpha-cyclodextrin with Active CAS RN 128446–33–3). However, even this designation can be uncertain, as in the singular case, in our experience, of a previously registered CAS RN 9009–54–5, propagated across the Internet and registered in both DSSTox and ChemID, which CAS has indicated was “created in error”, and thus was “deleted to zero” and “no longer exists” in the CAS database.

6 Note that structures containing both relative and absolute stereochemistry cannot be uniquely rendered in v2000 mol format; hence, v2000 sdf export is not supported in the current Dashboard. However, given that many publicly available software tools still import only v2000 mol or sdf files, SMILES and InChIs can be exported from the Dashboard and used to create compatible v2000 structures with the understanding that these may include degraded structures.

7 Note that the 1,181 name conflicts detected between DSSTox_V1 names and EPA SRS “No structures” content were considered unreliable indicators of conflict without a structure identifier for the comparison.

8 Note, a simple DSSTox Structure-Browser tool was provided previously in association with the original DSSTox website but was retired with the DSSTox website in 2015; up until that time, it represented the only structure-searching tool available on EPA’s public website.

9 A suspected error in the stereo form of a structure for CAS RN 527–09–3 (Absolute stereo was missing) was reported by a DSSTox curator, CAS reviewed the report and later added “Absolute stereo” to the structure, communicating back to us on 5/15/2017 about the addition to the structure.

10 An example is DTXSID30873022, which was previously registered with NOCAS_873022 but later appeared as registered in the CAS database with CAS RN 1135335–98–6 and the “Source of the Registration” listed as DSSTox.
