# Supplementary material for: EPA’s DSSTox database: History of development of a curated chemistry resource supporting computational toxicology research
Source: Comput Toxicol. Author manuscript; Available in PMC 2021 Jan 7. (PMC7787967; doi:10.1016/j.comtox.2019.100096)

EPA's DSSTox Chemical Structure Database: A curated chemistry resource supporting computational toxicology research (Grulke et al.)

Supplemental Material:

All websites accessed on 3/6/2019.

Example 1. PubChem record with multiple CAS RN and names synonyms assigned to "2-Octene". The trans (E) form of 2-Octene structure is shown, but three forms are listed as synonyms (cis, trans, mixture of cis/trans). DSSTox assigns each of these three forms to different substance records, each with its own substance name, CAS RN and structure.

https://pubchem.ncbi.nlm.nih.gov/compound/5364448

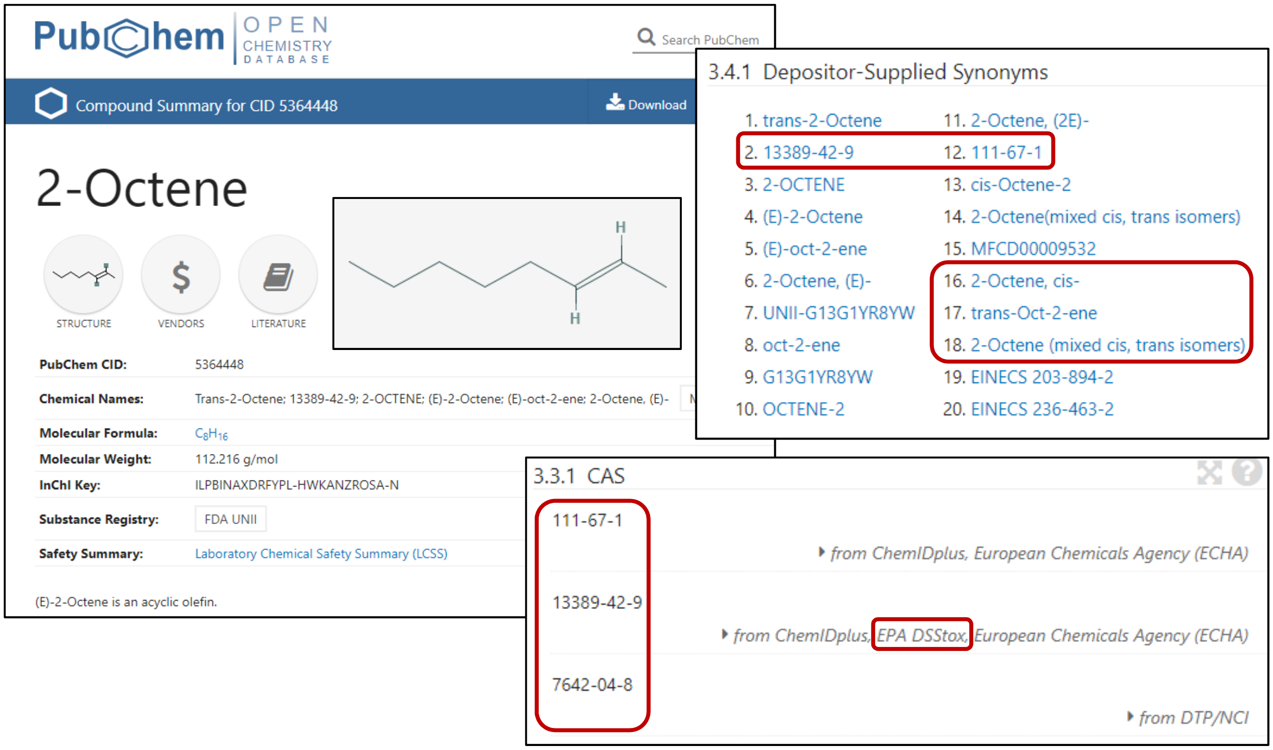

Example 2. PubChem and ChemID records with incorrect metal assigned to structure.

https://www.ncbi.nlm.nih.gov/pccompound?term=104199-51-1~%5Bsynonym%5D

PubChem Compound | PubChem Compound ▾ | 104199-51-1~[synonym]

Create alert Limits Advanced

Summary ▾ Sort by Default order ▾ Send to: ▾

Search results

Items: 2

1.

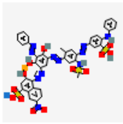

104199-51-1; Ferrate(2-), [3-(hydroxy-.kappa.O)-4-[[2-(hydroxy-.kappa.O)-4-hydroxy-5-[[2-methyl-5-[(methylsulfonyl)amino]-4-[[4-(phenylamino)-3-sulfophenyl]azo]phenyl]azo]-3-(phenylazo)phenyl]azo-.kappa.N1]-7-nitro-1-naphthalenesulfonato(4-)]-, sodium hydrogen; Ferrate(2-), [3-(hydroxy-.kappa.O)-4-[2-[2-(hydroxy-.kappa.O)-4-hydroxy-5-[2-[2-methyl-5-[(methylsulfonyl)amino]-4-[2-[4-(phenylamino)-3-sulfophenyl]diazenyl]phenyl]diazenyl]-3-(2-phenyldiazenyl)phenyl]azo-.kappa.N1]-7-nitro-1-naphthalenesulfonato(4-)]-, sodium hydrogen (1:1:1)

MW: 1073.797 g/mol MF: $C_{42}H_{32}FeN_{11}NaO_{13}S_3$

IUPAC name: sodium;4-[[5-[[4-[(4-anilino-3-sulfophenyl)diazenyl]-5-(meth...

Create Date: 2015-09-17

CID: 91867692

Summary Similar Compounds Same Parent, Connectivity Mixture/Component Compounds

2.

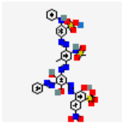

104199-51-1; Ferrate(2-), (3-(hydroxy-kappaO)-4-((2-(hydroxy-kappaO)-4-hydroxy-5-((2-methyl-5-((methylsulfonyl)amino)-4-((4-(phenylamino)-3-sulfophenyl)azo)phenyl)azo)-3-(phenylazo)phenyl)azo-kappaN1)-7-nitro-1-naphthalenesulfonato(4-))-, sodium hydrogen; Ferrate(2-), (3-(hydroxy-kappaO)-4-(2-(2-(hydroxy-kappaO)-4-hydroxy-5-(2-(2-methyl-5-((methylsulfonyl)amino)-4-(2-(4-(phenylamino)-3-sulfophenyl)diazenyl)phenyl)diazenyl)-3-(2-phenyldiazenyl)phenyl)azo-kappaN1)-7-nitro-1-naphthalenesulfonato(4-))-, sodium hydrogen (1:1:1) ...

MW: 1079.482 g/mol MF: $C_{42}H_{30}CuN_{11}NaO_{13}S_3$

IUPAC name: copper;sodium;4-[[5-[[4-[(4-anilino-3-sulfonatophenyl)diazen...

Create Date: 2007-07-03

CID: 16131308

Summary Similar Compounds Same Parent, Connectivity Mixture/Component Compounds

https://chem.nlm.nih.gov/chemidplus/rn/104199-51-1

TOXNET > ChemIDplus > Substance

Registry Number ▾ | equals ▾

ChemIDplus

A TOXNET DATABASE

Lite • Browse • Advanced

Start New Query | Modify Query | Search History

Substance Name: Ferrate(2-), (3-(hydroxy-kappaO)-4-(2-(2-(hydroxy-kappaO)-4-hydroxy-5-(2-(2-methyl-5-((methylsulfonyl)amino)-4-(2-(4-(phenylamino)-3-sulfophenyl)diazenyl)phenyl)diazenyl)-3-(2-phenyldiazenyl)phenyl)azo-kappaN1)-7-nitro-1-naphthalenesulfonato(4-))-, sodium hydrogen (1:1:1)

RN: 104199-51-1

InChIKey: PTXDUWIFJGIUPT-GROFPTGNSA-K

Structure Descriptors

InChI

1S/C42H33N11O13S3.Cu.Na/c1-23-17-32(47-45-26-13-16-30(38(18-26)69(64,65)66)43-24-9-5-3-6-10-24)33(52-67(2,59)60)20-31(23)46-48-34-21-35(42(56)40(41(34)55)51-44-25-11-7-4-8-12-25)49-50-39-28-15-14-27(53(57)58)19-29(28)37(22-36(39)54)68(61,62)63;;/h3-22,43,52,54-56H,1-2H3,(H,61,62,63)(H,64,65,66);;/q;+2;+1/p-3/b47-45+,48-46+,50-49+,51-44+;;

Download

InChIKey

PTXDUWIFJGIUPT-GROFPTGNSA-K

Search the web for this InChIKey

Smiles

[Cu+2].S([O-])(=O)(=O)c1cc(\N=N\c2c(cc(\N=N\c3cc(\N=N\c4c5c(cc(cc5)[N+]([O-])=O)c(cc4[O-])S([O-])(=O)=O)c(c(\N=N\c4ccccc4)c3O)[O-])c(C)c2)NS(C)(=O)=O)ccc1Nc1ccccc1.[Na+]

Download

Example 3. Sample public supplier website listing both the correct structure-name-CAS and a mismapped synonym-structure-CAS.

http://www.synquestlabs.com/product/id/72359.html

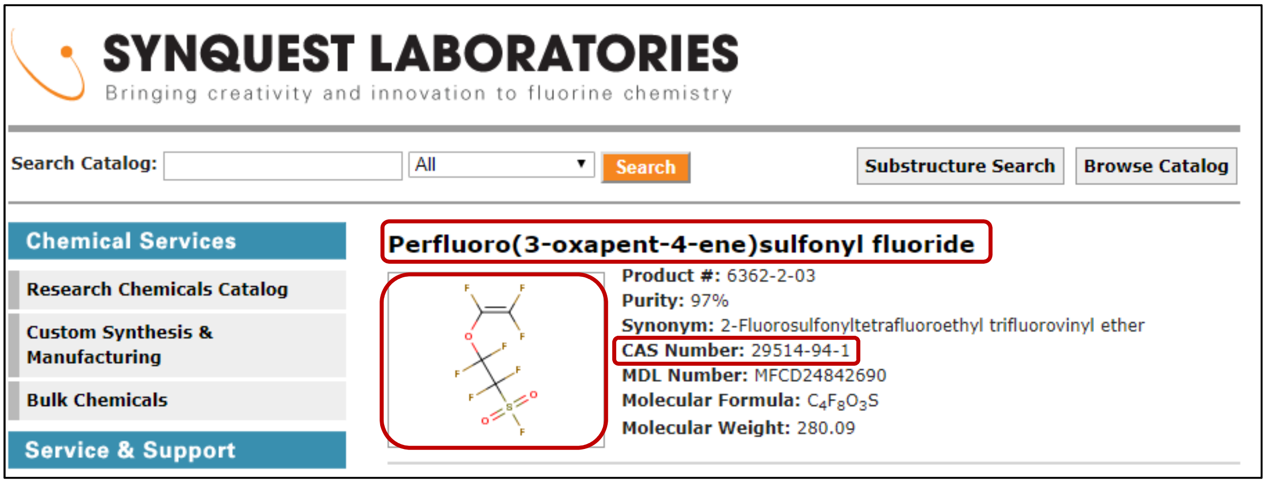

http://www.synquestlabs.com/product/id/27815.html

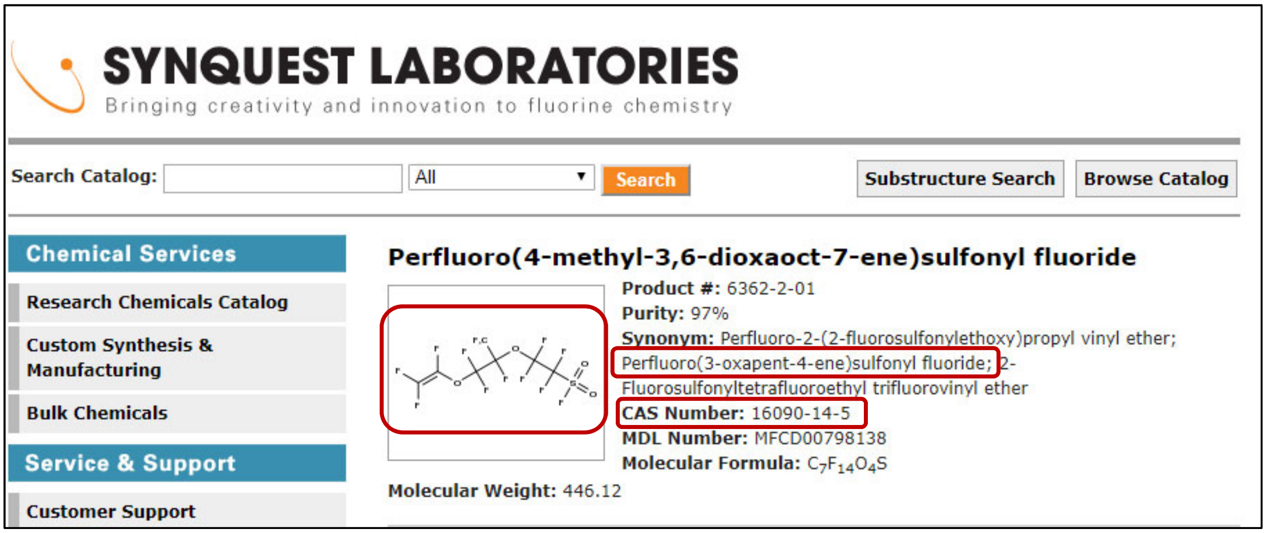

Example 4. Sample DSSTox substance record with ambiguous synonyms and how they are mapped within DSSTox, also showing instance of mapped deleted CAS RN.

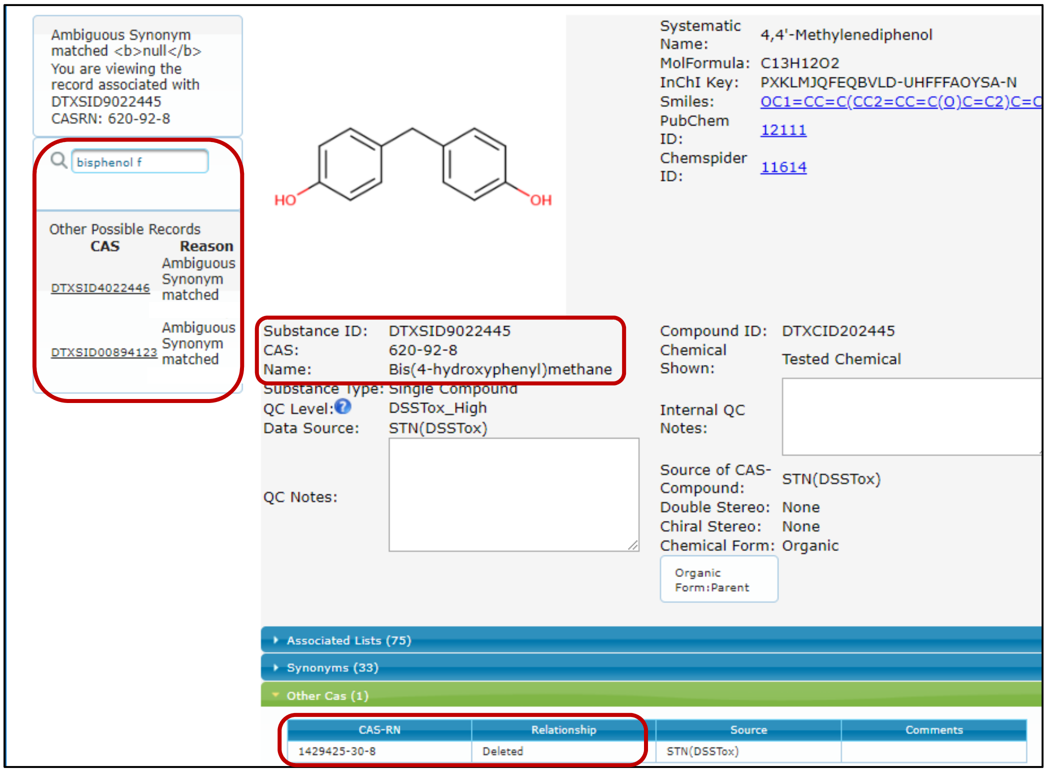

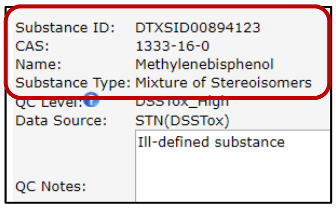

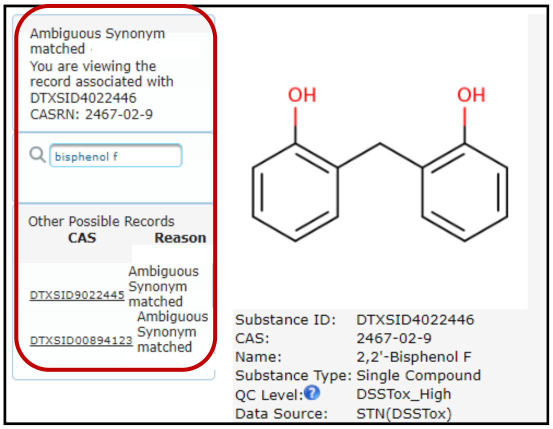

Example 5: Sample relationship mappings in DSSTox for mixtures mapped to a component.

https://comptox.epa.gov/dashboard/dsstoxdb/results?search=DTXSID8020339#related-substances

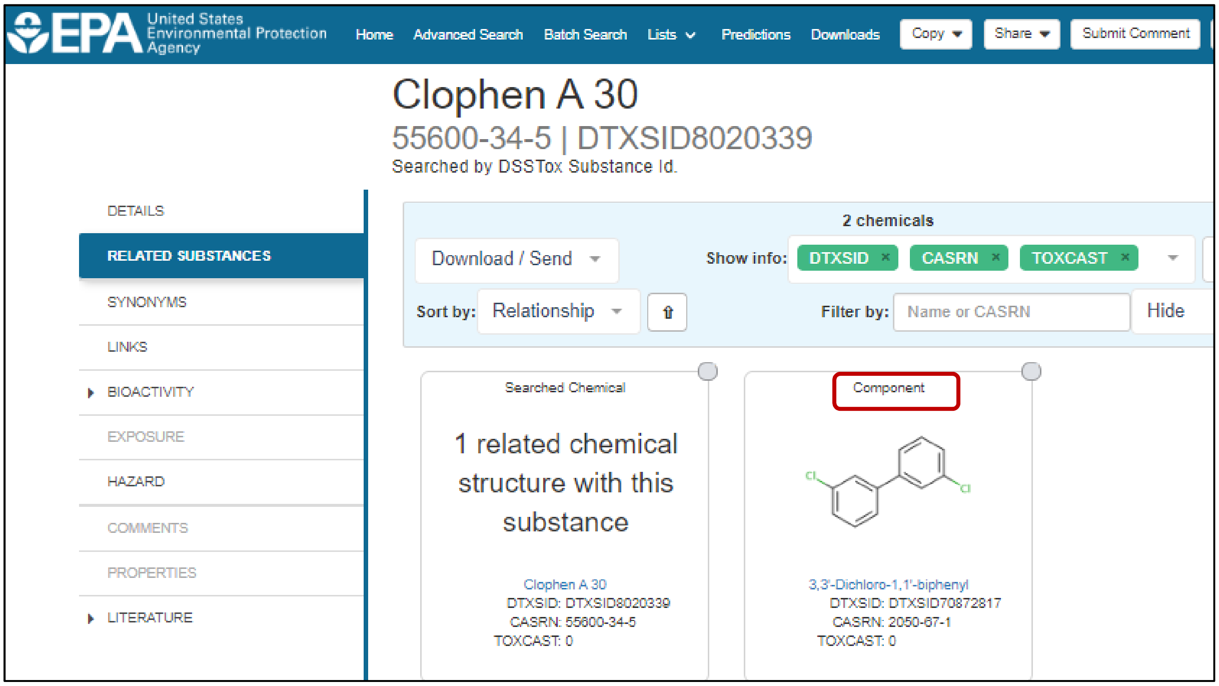

https://comptox.epa.gov/dashboard/dsstoxdb/results?search=DTXSID7057864#related-substances

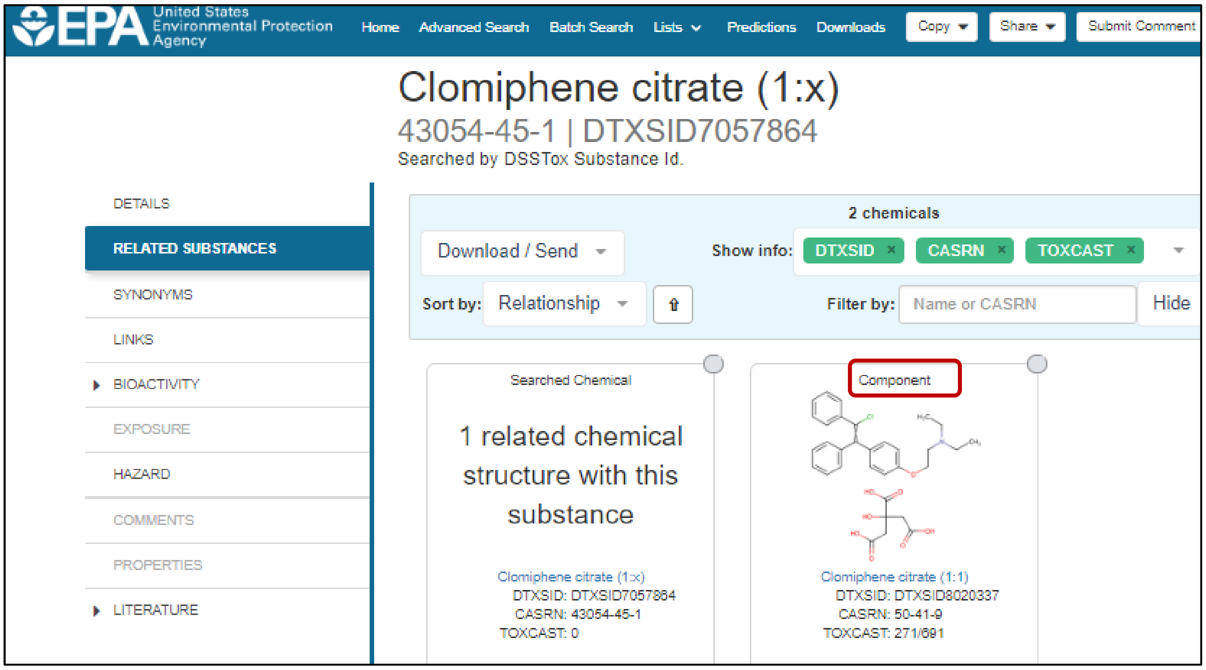

Example 6: Sample relationship mappings in DSSTox for polymer mapped to a monomer, also showing representative structures provided for the non-structurable record on 2 public websites.

https://comptox.epa.gov/dashboard/dsstoxdb/results?search=DTXSID5025071#related-substances

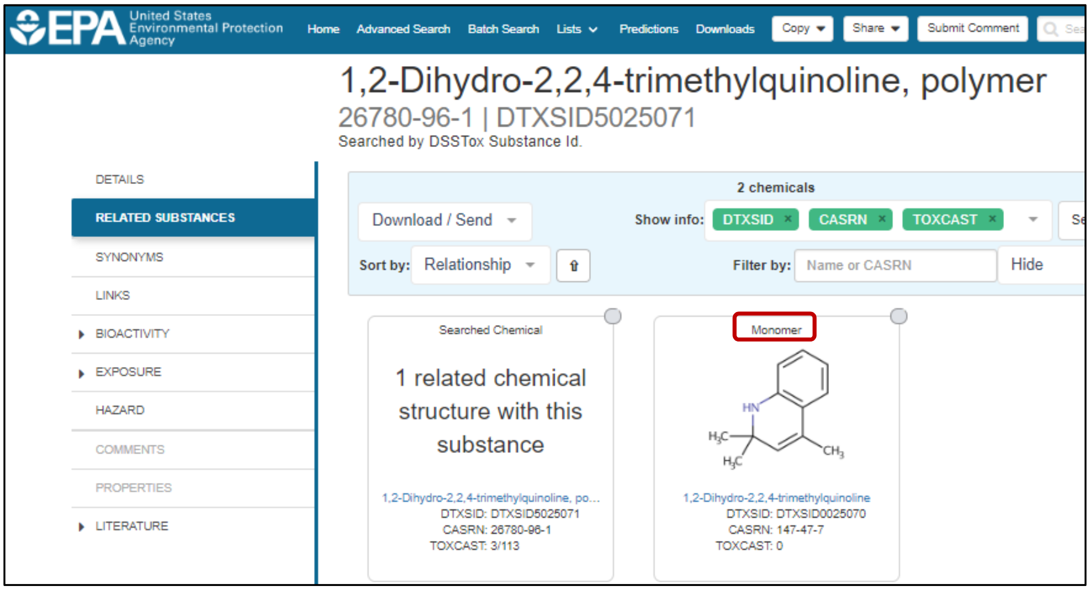

https://pubchem.ncbi.nlm.nih.gov/compound/8981#section=2D-Structure

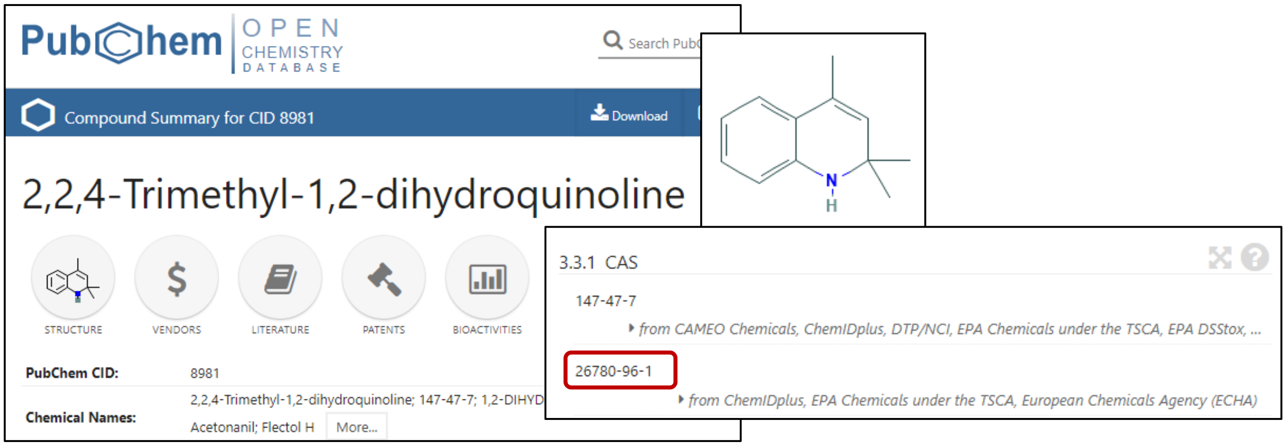

https://chem.nlm.nih.gov/chemidplus/rn/25703-79-1

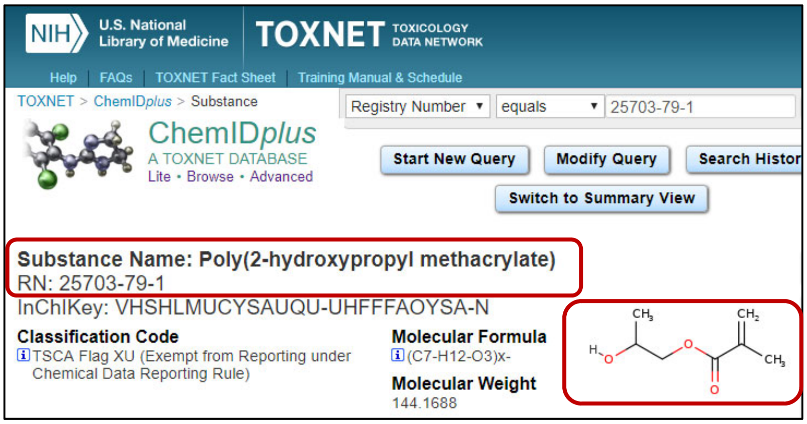

Example 7. Two sample DTXSID structures with advanced relative stereochemistry supported by v3000 mol files that are mapped by PubChem to v2000 structures in which the relative stereochemistry information is lost.

https://comptox.epa.gov/dashboard/DTXSID6020561

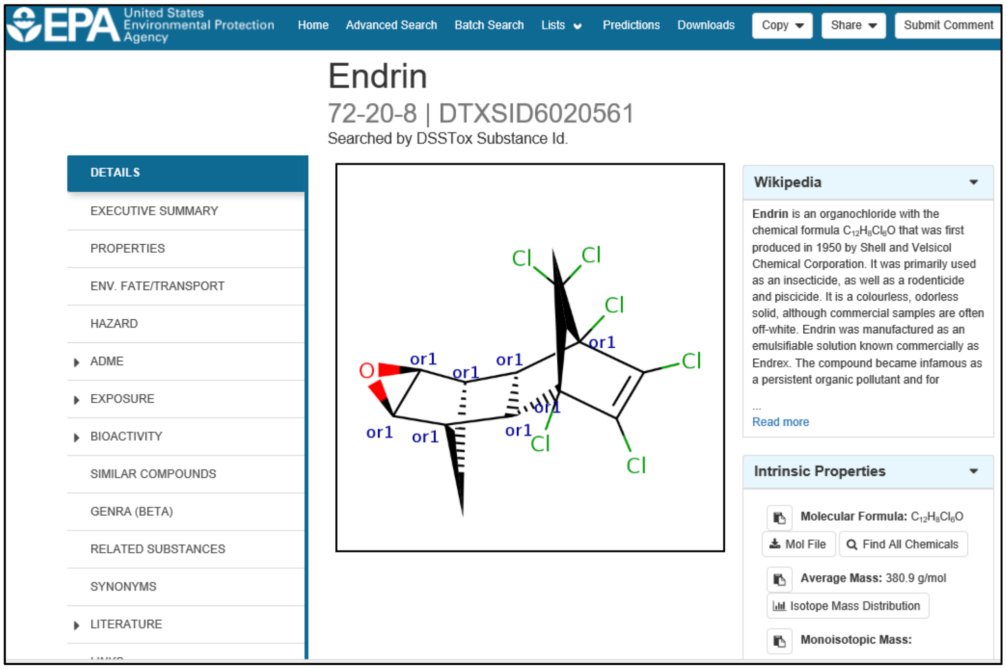

https://pubchem.ncbi.nlm.nih.gov/compound/12358480#section=2D-Structure

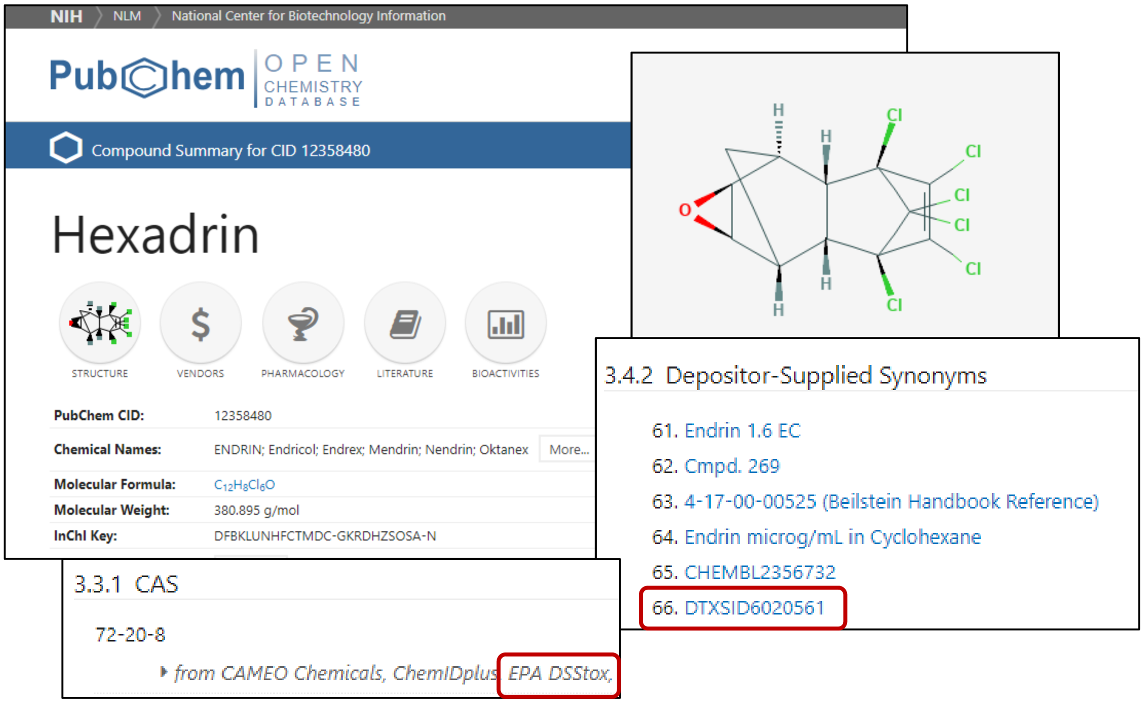

https://comptox.epa.gov/dashboard/dsstoxdb/results?search=DTXSID4023466

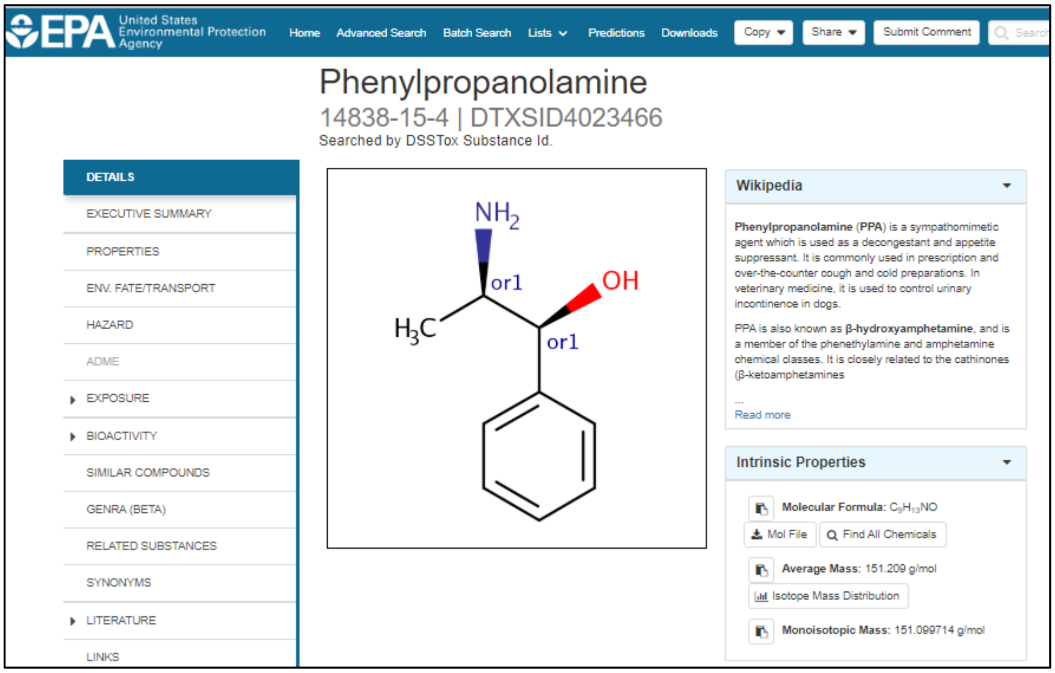

https://pubchem.ncbi.nlm.nih.gov/compound/26934#section=Depositor-Supplied-Synonyms

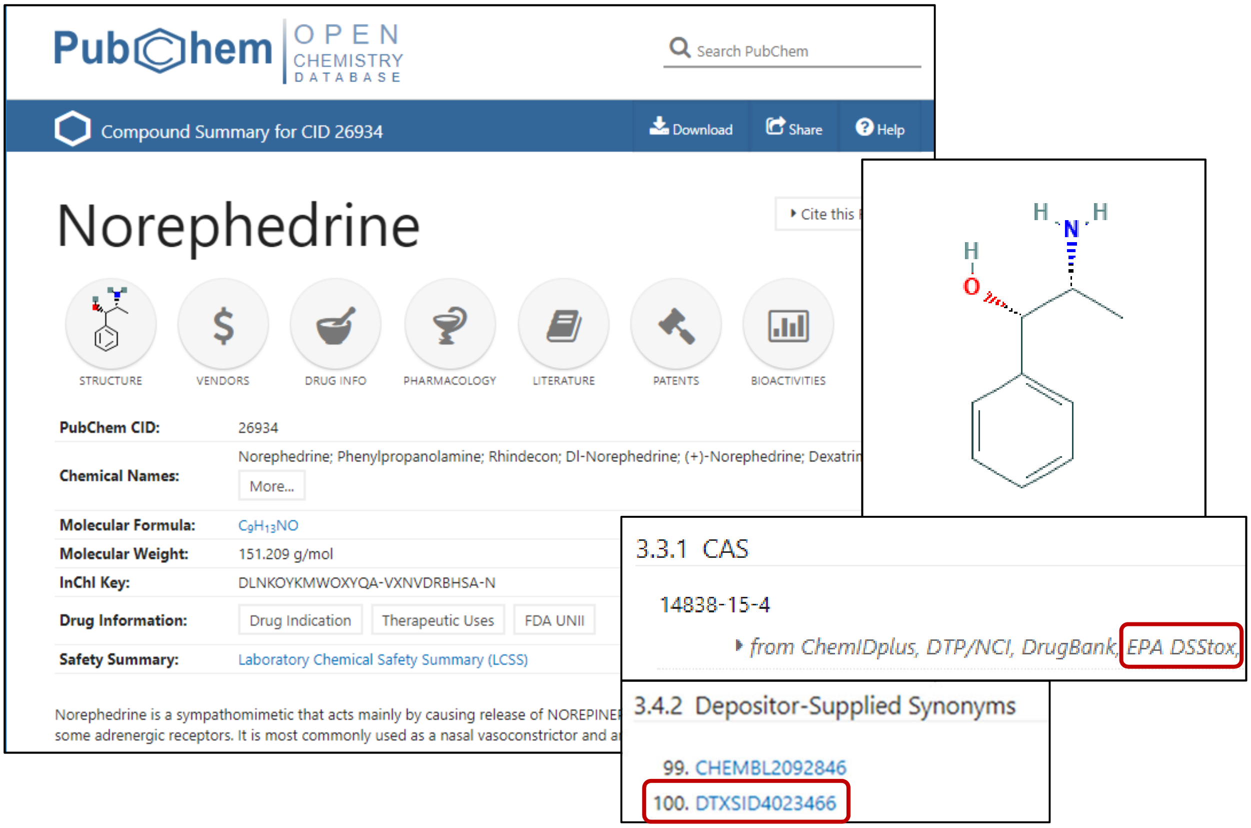

Supplement: Supplement1 [file NIHMS1647193-supplement-Supplement1.pdf]
